# Copper Cocatalyst Modulated Radical Generation for Selective Heterogeneous Photosynthesis of α-Haloketones

**DOI:** 10.1021/acscatal.2c05189

**Published:** 2022-12-15

**Authors:** Feiyu Han, Dongsheng Zhang, Sofia Salli, Jiani Ye, Yongwang Li, Federico Rosei, Xiao-Dong Wen, Hans Niemantsverdriet, Emma Richards, Ren Su

**Affiliations:** † Soochow Institute for Energy and Materials Innovations (SIEMIS), Soochow University, Suzhou, Jiangsu 215006, China; ‡ SynCat@Beijing, Synfuels China Technology Co. Ltd., Leyuan South Street II, No.1, Huairou, Beijing 101407, China; § School of Chemistry, Cardiff University, Park Place, Cardiff CF10 3AT, U.K.; ∥ State Key Laboratory of Coal Conversion, Institute of Coal Chemistry, CAS, Taiyuan 030001, China; ⊥ Center for Energy, Materials and Telecommunications, Institut National de la Recherche Scientifique, 1650 Boulevard Lionel-Boulet, Varennes, Québec J3X 1P7, Canada; # SynCat@DIFFER, Syngaschem BV, HH Eindhoven 6336, The Netherlands

**Keywords:** α-haloketones, heterogeneous photocatalysis, oxidative halogenation, oxygen radicals, halogen radicals

## Abstract

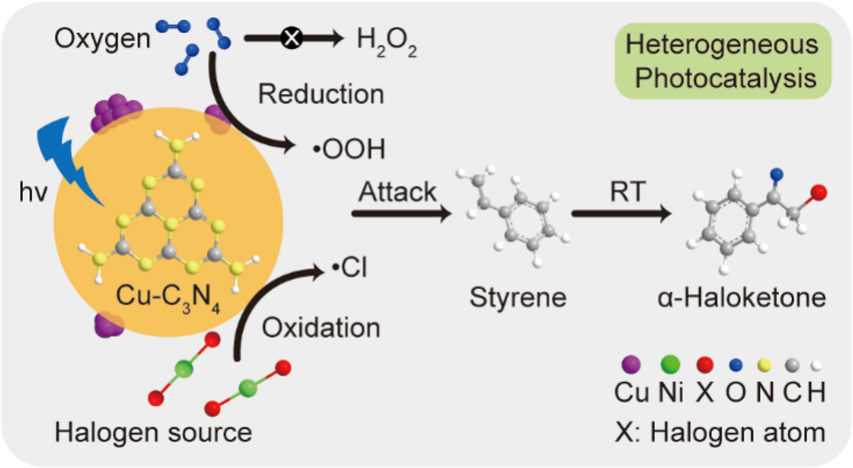

The α-haloketones are important precursors for synthetic chemistry and pharmaceutical applications; however, their production relies heavily on traditional synthetic methods via halogenation of ketones that are toxic and environmentally risky. Here, we report a heterogeneous photosynthetic strategy of α-haloketone production from aromatic olefins using copper-modified graphitic carbon nitride (Cu–C_3_N_4_) under mild reaction conditions. By employing NiX_2_ (X = Cl, Br) as the halogen source, a series of α-haloketones can be synthesized using atmospheric air as the oxidant under visible-light irradiation. In comparison with pristine carbon nitride, the addition of Cu as a cocatalyst provides a moderate generation rate of halogen radicals and selective reduction of molecular oxygen into ^•^OOH radicals, thus leading to a high selectivity to α-haloketones. The Cu–C_3_N_4_ also exhibits high stability and versatility, rendering it a promising candidate for solar-driven synthetic applications.

The α-haloketones are essential intermediates in synthetic chemistry and pharmaceutical manufacturing.^1−3^ Traditional synthesis of α-haloketones is realized by halogenation of the C–H bond of the corresponding ketones.^4^ These reactions exhibit high performance and decent selectivity; however, the need for harsh reaction conditions and toxic elemental halogens (i.e., Cl_2_, Br_2_) or organic halogens (i.e., CH_2_Cl_2_, *N*-bromosuccinimide (NBS), *N*-halosuccinimide (NCS)) as the halogen sources poses high demands toward the consideration of safety regulations and environmental protection.^5−10^ Direct conversion of olefins to the corresponding α-haloketones by employing oxidants and inorganic halogen salts is an eco-friendly, one-step process,^11^ yet the challenges in choosing suitable precursors, selectivity control, and the limited substrate scope available through this route restrict its large-scale application. Additionally, the evolution of unwanted elemental halogens during the process is inevitable due to the use of strong oxidants (i.e., K_2_S_2_O_8_).

The synthesis of α-haloketones by photocatalytic oxidative halogenation of olefins is an alternative approach that has been developed in recent years.^12−14^ It possesses several advantages compared to conventional synthetic approaches. First, the electrons generated upon irradiation can activate molecular oxygen to oxidize the olefins into corresponding ketones, thus avoiding the use of strong oxidants. Second, the mild reaction conditions of photocatalysis render the conversion of olefins with susceptible functional groups applicable. Nevertheless, by tuning the oxidation power of the photocatalysts, it is possible to achieve halogenation of the photogenerated ketones without the formation of elemental halogen during the whole reaction course, rendering it a mild process that is ideal for applications with a high demand of environmental preservation. Wang et al. report the synthesis of a series of α-haloketones from olefins under visible-light irradiation using Ru(bpy)_3_Cl_2_ as the sensitizer, PhI(OAc)_2_ as the catalyst, and organic halides (CH_3_X) as the halogen source.^15^ Meanwhile, the hazardous CH_3_X halogen source can be replaced by inorganic salts (KBr and KCl) when catalyzed by metal halides (FeX_3_) in the presence of *p*-toluenesulfonic acid (TsOH) under visible-light irradiation, though the need for an oxygen-rich atmosphere is a major setback.^16,17^ To date, homogeneous photocatalytic systems rely intensively on expensive catalysts, special additives, and complicated reaction conditions, limiting the photocatalytic approach for wide-scale application. Therefore, the development of a selective and efficient heterogeneous photocatalyst for the synthesis of α-haloketones using nontoxic inorganic halogen sources under ambient conditions is desirable.

Three criteria need to be considered to construct a promising heterogeneous photocatalyst from a catalytic perspective (Scheme 1). First, the photocatalyst should be able to create suitable oxygen-based and halogen radical species with moderate kinetics to attack the olefins. However, a rapid formation rate of oxygen radical species will lead to unwanted dissociation of the reactant molecule and over oxidation of the α-haloketones to aldehydes, whereas too high a concentration of halogen radical species tends to produce dihalogenated compounds and the evolution of elemental halogen. Second, the reduction of molecular oxygen has often been ignored. The identity and kinetics of photogenerated oxygen radical species govern the overall rate and selectivity of the photocatalytic process. Here, the superoxide radical (^•^OOH) is favored, as other oxygen radicals (i.e., ^•^OH) are associated with the formation of unwanted carbonyls.^18^ Lastly, the reduction product of O_2_ (i.e., H_2_O) needs to desorb from the surface of the photocatalyst rapidly to complete the catalytic cycle. A series of recent studies show that the aforementioned factors can be manipulated by employing metal cocatalysts supported on the photocatalysts,^19−23^ thus leading to efficient and selective photosynthesis,^24−27^ indicating that the selective oxidative halogenation of olefins may be achieved by such a strategy. The graphitic carbon nitride (g-C_3_N_4_) is the ideal photocatalyst support, owing to its suitable band positions and versatility for multiple organic transformations.^28^

**Scheme 1:**
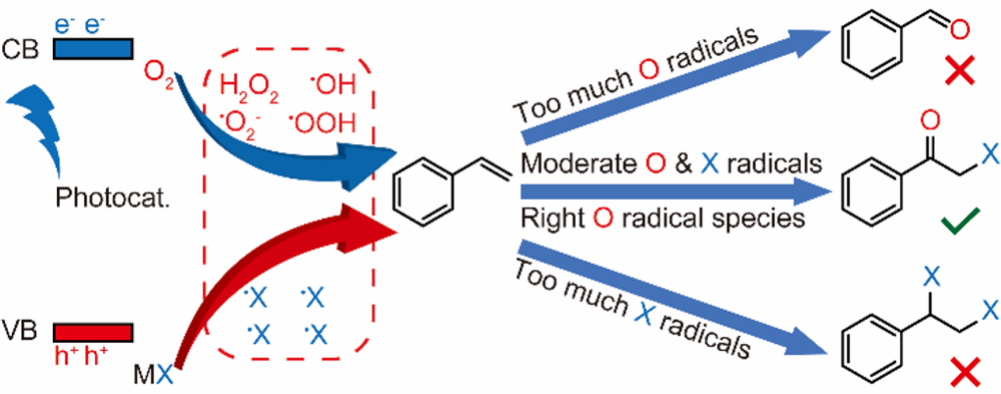
Reaction Pathways of Photocatalytic Styrene Conversion Governed by Radical Species and Quantities

Herein, we report a facile synthesis of α-haloketones from oxidative halogenation of aromatic olefins by employing a copper-modified graphitic carbon nitride (Cu–C_3_N_4_) photocatalyst using NiCl_2_ as the halogen source under visible-light irradiation and ambient conditions. The Cu–C_3_N_4_ photocatalyst presents an optimum formation of superoxide radicals (^•^OOH), a moderate evolution of halogen radicals (^•^X), and a weak adsorption of photogenerated water, thus resulting in an optimum selectivity to α-haloketones compared to other metal-decorated C_3_N_4_. The potential of Cu–C_3_N_4_ for application in terms of stability and substrate scope is also discussed.

## Catalyst Characterizations

**Figure 1:**
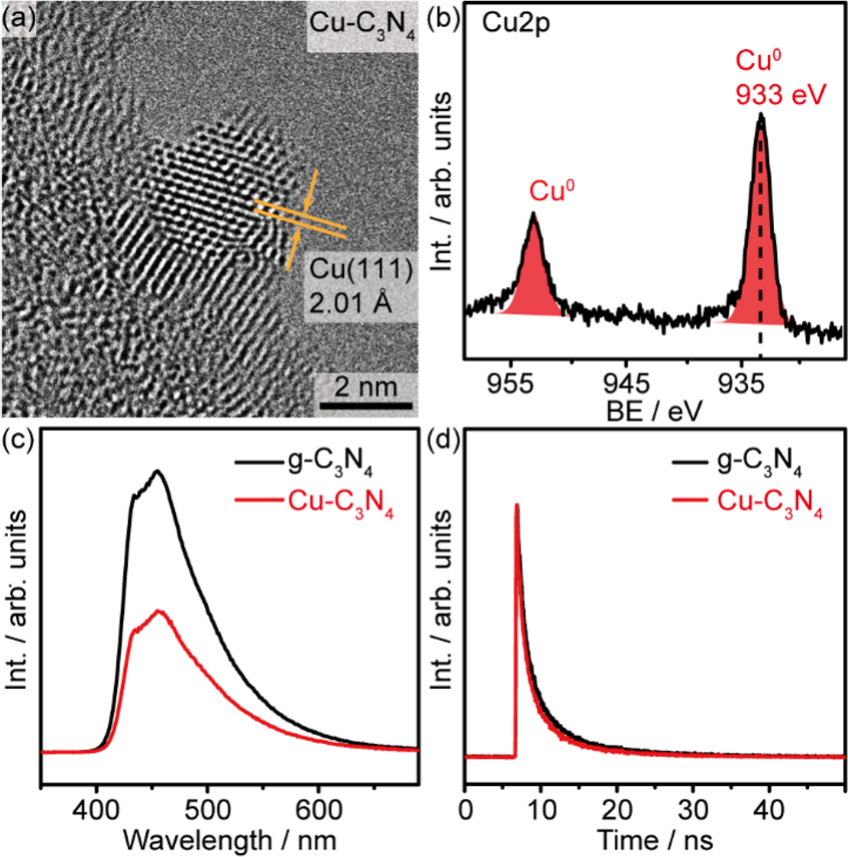
Characterization of the photocatalyst. (a) and (b) TEM image and Cu 2p XPS spectra of the Cu–C_3_N_4_. (c) and (d) Steady-state and transient fluorescence spectra of Cu–C_3_N_4_ and g-C_3_N_4_.

The pristine g-C_3_N_4_ photocatalyst is synthesized via pyrolysis of urea, and Cu is loaded on the g-C_3_N_4_ via a conventional photodeposition method in an isopropanol–water solution under deaerated conditions (Supporting Information Experimental Procedures and Figure S1). The inductively coupled plasma-atomic emission spectrometry (ICP-AES) analysis reveals that the loading of Cu is ∼0.15 wt % (Table S1 in the Supporting Information). Transmission electron microscopy (TEM) imaging of a single Cu nanoparticle (NP) of the as-synthesized photocatalyst suggests that polycrystalline metallic copper with exposed (111) facets (*d* = 2.01 Å) is anchored on the edge of the g-C_3_N_4_ (Figure 1a). X-ray photoelectron spectroscopy (XPS) analysis of the pristine g-C_3_N_4_ and Cu–C_3_N_4_ confirms the purity of the catalyst (Figure S2 in the Supporting Information). The region-of-interest Cu 2p spectrum suggests that Cu is in its metallic state (narrow peak at 933 eV, Figure 1b). The absence of satellite features at ∼943–945 eV indicates that no oxide species are present. The powder X-ray diffraction (PXRD) patterns of both pristine g-C_3_N_4_ and Cu–C_3_N_4_ are identical, displaying the characteristic diffraction peaks of (100) and (002) facets of g-C_3_N_4_, implying a negligible destruction of the nanocrystalline structure caused by the Cu loading (Figure S3 in the Supporting Information). The Cu (111) diffraction peak is nondetectable due to the low loading of Cu. Diffuse reflectance spectroscopy (DRS) shows that the pristine g-C_3_N_4_ and Cu–C_3_N_4_ exhibit a similar band gap (∼2.8 eV) and light absorption properties (Figure S3 in the Supporting Information). The steady-state photoluminescence (PL) spectroscopy shows a characteristic asymmetric emission peak (∼440 nm) for g-C_3_N_4_ (Figure 1c), confirming that the radiative recombination mainly takes place at trap states with energy levels close to the conduction band minimum (CBM) or the valence band maximum (VBM). The loading of Cu metal NPs only reduces the intensity of the emission peak, suggesting an enhanced charge separation without altering the electronic structure of the g-C_3_N_4_. However, time-resolved fluorescence spectroscopy suggests that the presence of Cu NPs does not influence the radiative recombination kinetics of photogenerated charge carriers (Figure 1d).^29^

## Performance Evaluation

Styrene (**1a**) is employed as the model compound to evaluate the photocatalytic performance of a series of metal-decorated g-C_3_N_4_ and the effect of metal chloride and solvent under 410 nm irradiation at room temperature (RT) and atmospheric conditions (Table 1). The conversion and selectivity of the reaction were determined by gas chromatography (GC) and gas chromatography–mass spectrometry (GC-MS) using standard compounds (see Experimental Procedures in the Supporting Information). The Cu–C_3_N_4_ photocatalyst fully converts styrene into 2-chloroacetophenone (**2a**) with high selectivity (80%) in an isopropanol–ethyl acetate solvent (1:9 v/v) with nickel chloride hexahydrate as the Cl source (Table 1, entry 1). In comparison, the pristine g-C_3_N_4_ and other metal (Ag, Fe, Ni)-decorated g-C_3_N_4_ show poor selectivity to **2a** (Table 1, entries 2–5), indicating the essential role of Cu as the cocatalyst. GC-MS analysis reveals that the alternative g-C_3_N_4_ photocatalysts tend to produce 1,2-dichloroethylbenzene and 2-chloro-1-phenylethanol as byproducts (Figure S4 in the Supporting Information). Among the tested chlorine sources, nickel chloride hexahydrate outperforms the anhydrous nickel chloride (Table 1, entry 6) due to a better solubility in organic solvent. Interestingly, ferric chloride, sodium chloride, potassium chloride, and copper chloride all present poor performance in photocatalytic halogenative oxidation of styrene (Table 1, entries 7–10). This can be ascribed to the suitable redox potential of Ni^2+^/Ni^0^ (Ni^2+^/Ni = −0.257 V vs RHE) that allows its reduction by the conduction band electron of the excited g-C_3_N_4_. The solvent also significantly influences the selectivity of the reaction. A poor selectivity is observed in the absence of isopropanol due to the oxidative dissociation of **2a** into benzaldehyde (Table 1, entry 11), indicating that isopropanol as a hydrogen donor can provide sufficient H atoms to avoid overoxidation of the generated **2a**. No deuterated products are observed when CH_3_CH_2_OD was used as the hydrogen donor (Figure S5 in the Supporting Information), confirming that the abstracted D atoms convert into D_2_O by reacting with molecular oxygen. A similar phenomenon is observed when other aprotic solvents are use (Table 1, entries 12–14). Acetophenone becomes the dominant product in pure protic solvents due to the dehalogenation of photogenerated α-haloketones (isopropanol and ethanol, Table 1, entries 15 and 16). Additionally, control experiments show that the reaction is indeed a photocatalytic process (entry 4, Table S2 in the Supporting Information).

**Table 1:**
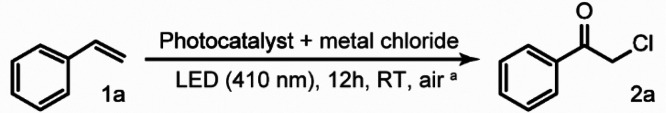
Screening of Catalysts and Reaction Conditions: Photosynthesis of 2-Chloroacetophenone from Styrene^a^

Entry	Catalysts	Chloride	Solvent	Con./%^b^	Sel./%^b^
1	Cu–C_3_N_4_	NiCl_2_·6H_2_O	EtOAc-*i*PrOH^c^	99	80
2	g-C_3_N_4_	NiCl_2_·6H_2_O	EtOAc-*i*PrOH	99	52
3	Ag–C_3_N_4_	NiCl_2_·6H_2_O	EtOAc-*i*PrOH	83	58
4	Fe–C_3_N_4_	NiCl_2_·6H_2_O	EtOAc-*i*PrOH	99	47
5	Ni–C_3_N_4_	NiCl_2_·6H_2_O	EtOAc-*i*PrOH	88	57
6	Cu–C_3_N_4_	NiCl_2_	EtOAc-*i*PrOH	77	69
7	Cu–C_3_N_4_	FeCl_3_	EtOAc-*i*PrOH	22	11
8	Cu–C_3_N_4_	NaCl	EtOAc-*i*PrOH	20	17
9	Cu–C_3_N_4_	KCl	EtOAc-*i*PrOH	20	20
10	Cu–C_3_N_4_	CuCl_2_	EtOAc-*i*PrOH	31	0
11	Cu–C_3_N_4_	NiCl_2_·6H_2_O	CH_3_CN	99	57
12	Cu–C_3_N_4_	NiCl_2_·6H_2_O	EtOAc	99	49
13	Cu–C_3_N_4_	NiCl_2_·6H_2_O	1,4-Dioxane	69	52
14	Cu–C_3_N_4_	NiCl_2_·6H_2_O	THF	93	63
15	Cu–C_3_N_4_	NiCl_2_·6H_2_O	*i*PrOH	99	0
16	Cu–C_3_N_4_	NiCl_2_·6H_2_O	EtOH	81	0

a Reaction conditions: styrene (8 mM), catalyst (50 mg), chlorine source (0.2 mmol), solvent (10 mL), RT, 1 bar air, 410 nm LED (30 mW cm^–2^) irradiation for 12 h.

b Conversion (Con.) and selectivity (Sel.) are determined by GC and GC-MS.

c 1 mL of isopropanol in 9 mL of ethyl acetate.

**Figure 2:**
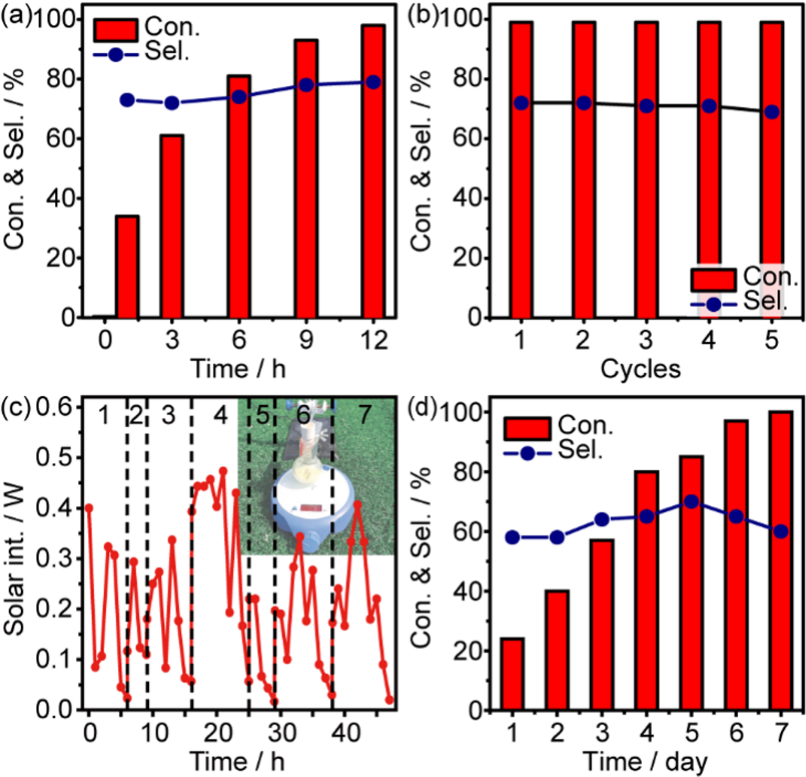
Photocatalytic performance. (a) Time-course study of photocatalytic styrene conversion to 2-chloromoacetophenone using Cu–C_3_N_4_. Reaction conditions: 8 mM styrene, 50 mg of catalyst, 0.2 mmol of NiCl_2_, and 1 mL of isopropanol in 9 mL of ethyl acetate under 1 bar air, irradiated under 410 nm LED (30 mW cm^–2^) at RT. (b) Stability of Cu–C_3_N_4_ in photocatalytic styrene conversion to 2-bromoacetophenone. Reaction conditions: 8 mM styrene, 10 mg of catalyst, 0.02 mmol of NiBr_2_, and 0.1 mL of isopropanol in 1.9 mL of ethyl acetate under 1 bar air, irradiated under 410 nm LED (30 mW cm^–2^) at RT. Each cycle is irradiated for 2 h during the stability test. (c) and (d) Recorded solar intensity and solar-driven styrene conversion using Cu–C_3_N_4_. The reaction was performed in Suzhou, China, from July 1, 2021 to July 8, 2021.

We have further investigated the heterogeneous photocatalytic oxidative addition of styrene employing Cu–C_3_N_4_ under optimized reaction conditions. A time-course analysis reveals that styrene is directly converted into 2-chloroacetophenone following pseudo-first-order kinetics (*k* = 0.31 h^–1^) within 12 h (Figure 2a). The selectivity to 2-chloroacetophenone remains at ∼80%, with benzaldehyde (∼4%), acetophenone (∼3%), 2-chloro-1-phenylethanol (∼7%), and dichloro-ethylbenzene (∼6%) as the major byproducts. A drop of the performance is observed with an increase of the styrene concentration, possibly due to its self-polymerization^30^ and the dehalogenation of α-haloketones (Figure S6 in the Supporting Information). The stability of Cu–C_3_N_4_ has also been evaluated (Figure 2b). The Cu–C_3_N_4_ photocatalyst presents a stable performance for five cycles by simply applying a DI water washing and HCl (100 mM) washing after each cycle. Additionally, the Cu–C_3_N_4_ photocatalyst is capable of utilizing solar energy to fully convert styrene into 2-chloroacetophenone with a reasonable selectivity (>60%, Figures 2c and 2d). The drop of the selectivity may be associated to the higher energy of the photons in the UV region, resulting in the formation of unwanted radicals and the generation of complicated byproducts. Though the fluctuation of irradiation intensity and unoptimized bulk reactor limits the performance, it still demonstrates the potential of solar photocatalysis for practical synthetic applications.

## Reaction Mechanisms

Since the photocatalytic oxidative halogenation of aromatic olefins involves the reduction of molecular oxygen and oxidation of a halogen anion, we have first examined the role of Cu NPs in the generation of reactive oxygen species (ROS) and halogen radicals using electron spin resonance (ESR).

**Figure 3:**
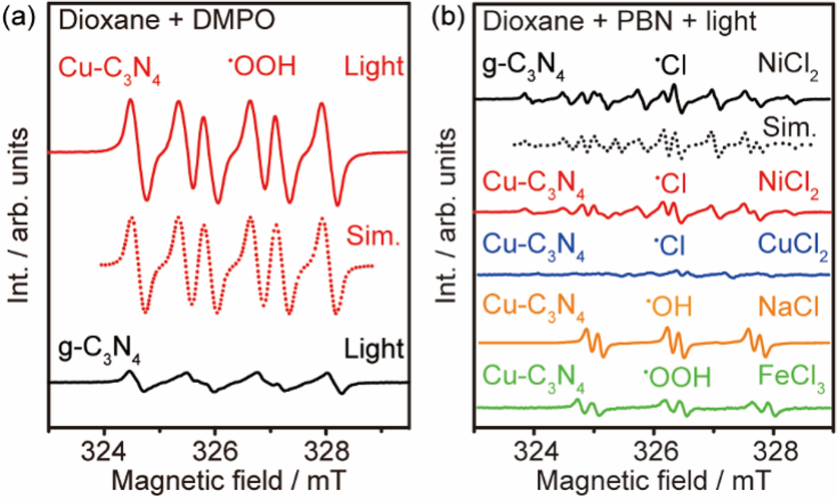
Cu-modulated evolution of radical species. (a) Evolution of oxygen radical species in the absence of metal chloride by Cu–C_3_N_4_ and g-C_3_N_4_ photocatalysts under irradiation using DMPO as the spin trap. Reaction conditions: 10 mg of catalyst and 20 mM DMPO in dioxane, irradiated at RT for 1.5 min. (b) Evolution of radical species with the presence of metal chloride by g-C_3_N_4_ and Cu–C_3_N_4_ photocatalysts under irradiation using PBN as the spin trap. Reaction conditions: 10 mg of catalyst, 0.02 mmol of metal chloride, and 20 mM PBN in dioxane, irradiated at RT for 1.5 min.

This is achieved by using 5,5-dimethyl-1-pyrroline *N*-oxide (DMPO) and phenylbutylnitrone (PBN) as spin traps under different reaction conditions (Figure 3 and Figure S7 in the Supporting Information). In the absence of NiCl_2_ (Figure 3a), both g-C_3_N_4_ and Cu–C_3_N_4_ generate oxygen radical species upon irradiation; however, the different spectra imply that distinct oxygen radical species are evolved in the presence of the different photocatalysts. The ESR spectrum recorded using the Cu–C_3_N_4_ photocatalyst contains only one component with relatively high intensity, assigned by simulation to the ^•^OOH radical (α[^1^H_β_] = 24.96, α[^1^H_γ_] = 3.37, and α[^14^N] = 38.15 MHz). In contrast, the complicated ESR spectrum of pristine g-C_3_N_4_ suggests that multiple oxygen radical species beyond ^•^OOH are evolved under irradiation. The weak intensity of the ESR spectrum indicates that either a slow generation or a rapid recombination kinetics of the oxygen radical species takes place on g-C_3_N_4_.

In the presence of NiCl_2_, both pristine g-C_3_N_4_ and Cu–C_3_N_4_ photocatalysts produce ^•^Cl radicals (α[^1^H] = 2.19, α[^14^N] = 35.51, and α[^35^Cl] = 18.53 MHz) upon irradiation (Figure 3b). Note that the pristine g-C_3_N_4_ shows a slightly higher ESR signal intensity due to a higher concentration of photogenerated chlorine radicals. However, this is not beneficial for the synthesis of α-haloketones, as a high concentration of ^•^Cl radicals leads to the formation of dichloro-substituted compounds according to the product analysis (Figure S4 in the Supporting Information). Noticeably, the identity of the metal chloride is also a crucial parameter that influences the photocatalytic reaction. The addition of CuCl_2_, NaCl, and FeCl_3_ only produces a trace amount of ^•^Cl radical in comparison with NiCl_2_. Instead, a detectable amount of hydroxyl radicals (^•^OH) and superoxide radicals (^•^OOH) are produced when NaCl and FeCl_3_ are used as the chlorine source, respectively. These results are in line with the catalytic performance, revealing that NiCl_2_ is the ideal chlorine source owing to its selective and mild generation of ^•^Cl radicals.

**Figure 4:**
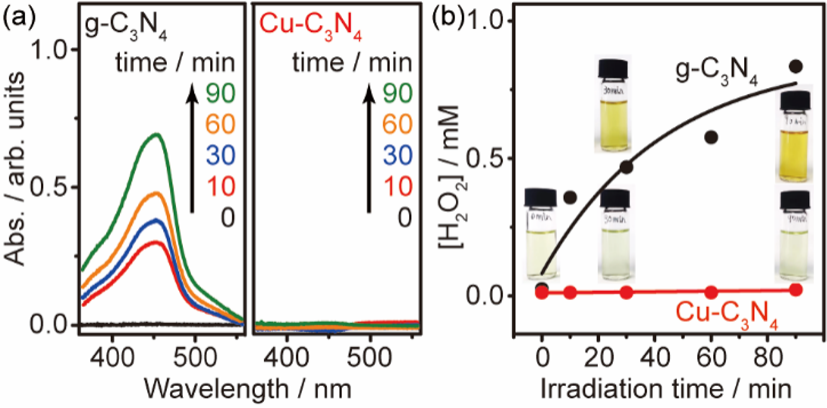
Cu-modulated evolution of H_2_O_2_. (a) UV–vis analysis of H_2_O_2_ formation using g-C_3_N_4_ and Cu–C_3_N_4_ under irradiation. (b) Kinetic analysis of H_2_O_2_ evolution. Reaction conditions: 50 mg of catalyst and 1 mL of isopropanol in 9 mL of ethyl acetate under ambient pressure, irradiated under 410 nm LED (30 mW cm^–2^) at RT.

We have further probed the evolution of H_2_O_2_ upon irradiation by UV–vis spectrometry titrated using a CuSO_4_:2,9-dimethyl-1,10-phenanthroline (DMP) solution (Figure 4a).^31,32^ A gradual increase of the absorption peak at 454 nm is observed for g-C_3_N_4_, implying the accumulation of photogenerated H_2_O_2_. Surprisingly, an absence of H_2_O_2_ is observed for Cu–C_3_N_4_ throughout the whole irradiation course. This is also evidenced by the color change of the solution (Figure S8 in the Supporting Information). Quantitative analysis further reveals that the formation of H_2_O_2_ on g-C_3_N_4_ follows pseudo first-order kinetics with a rate constant of 0.023 min^–1^, whereas the Cu–C_3_N_4_ inhibits the H_2_O_2_ evolution completely (Figure 4b).

**Figure 5:**
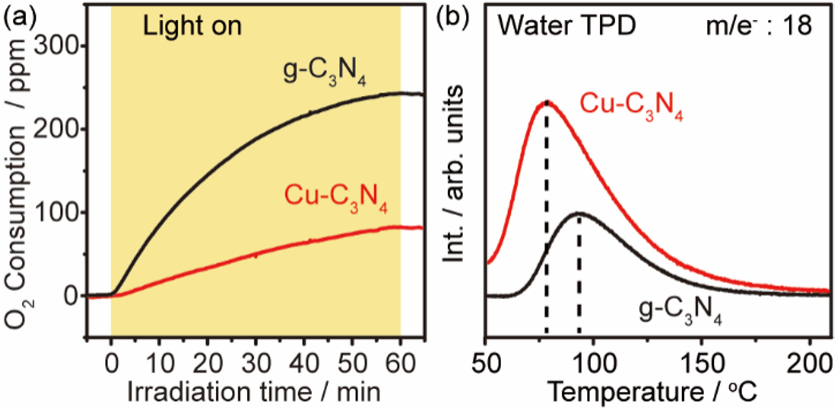
Cu-modulated O_2_ reduction and H_2_O desorption. (a) Oxygen consumption during photocatalytic conversion of styrene using g-C_3_N_4_ and Cu–C_3_N_4_. Reaction conditions: 15 mM styrene, 50 mg of catalyst, 0.2 mmol of NiCl_2_·6H_2_O, and 1 mL of isopropanol in 9 mL of ethyl acetate under 1 bar of atmosphere with 500 ppm of O_2_, irradiated under 410 nm LED (30 mW cm^–2^) at RT. (b) TPD of preadsorbed H_2_O on g-C_3_N_4_ and Cu–C_3_N_4_.

Interestingly, the pristine g-C_3_N_4_ exhibits a faster oxygen consumption rate (243 ppm·h^–1^) than that of Cu–C_3_N_4_ (82 ppm·h^–1^) under irradiation, as monitored using an oxygen sensor (Figure 5a and Figure S9 in the Supporting Information). This is in good agreement with the photocatalytic performance of pristine g-C_3_N_4_, which presents a fast conversion of styrene but a poor selectivity to **2a** compared to that of Cu–C_3_N_4_ under identical reaction conditions (Table 1, entry 2, and Figure S10 in the Supporting Information). In connection with the product analysis in photocatalytic performance tests and previous investigations,^33−35^ we propose that the faster oxygen consumption rate observed for pristine g-C_3_N_4_ generates ROS in a nonselective fashion (H_2_O_2_ and ^•^OOH), thus leading to the formation of unwanted byproducts. In comparison, the presence of Cu on g-C_3_N_4_ regulates the dissociation path of molecular oxygen to produce solely the ^•^OOH radical, thus resulting in a high selectivity to α-haloketones.

Additionally, the interaction of water (i.e., the final reduction product of O_2_) with the photocatalyst has been evaluated by temperature-programmed desorption (TPD, Figure 5b). While a desorption peak of water is observed at 77 °C for the Cu–C_3_N_4_, the pristine g-C_3_N_4_ presents a relatively stronger interaction of water as characterized by a desorption peak located at a higher temperature of 91 °C. This suggests that the water molecules generated during the photocatalytic synthesis of α-haloketones only need to overcome a small barrier to desorb from the surface of the Cu–C_3_N_4_ under ambient conditions, which is crucial to complete the catalytic cycle.

**Figure 6:**
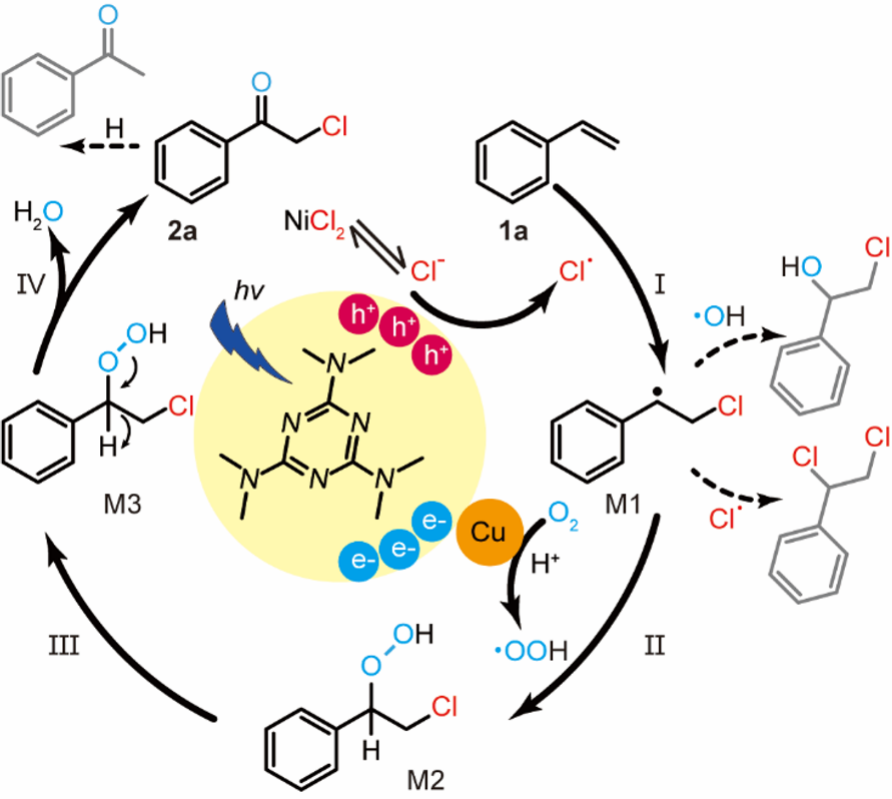
Proposed catalytic cycle and the role of Cu in modulating the generation of radical species. Side reactions and byproducts are indicated by the dashed lines and molecules in gray color.

A possible mechanism of the Cu–C_3_N_4_-promoted photocatalytic oxidative halogenation is proposed using styrene as the model compound and NiCl_2_ as the chlorine source (Figure 6). First, ^•^Cl radicals are created *via* the oxidation of chloride ions by the photogenerated holes from the Cu–C_3_N_4_ photocatalyst upon irradiation. Then the ^•^Cl radicals attack **1a** to generate the carbon-centered radical species (intermediate M1, step I). The M1 intermediate is further attacked by the ^•^OOH radical that is created *via* the reduction of molecular oxygen by the photogenerated electrons on the Cu cocatalyst to yield the intermediate M2 (step II). In step III, the metastable M2 is converted to intermediate M3 *via* a molecular restabilization process. Finally, the M3 releases a water molecule to generate **2a** (step IV).^17^ The byproducts, namely, 2-chlorophenylethanol and dichloroethylbenzene, are generated from ^•^OH and ^•^Cl radicals attacking the intermediate M1, respectively. The acetophenone is produced *via* the hydro-dechlorination of the photogenerated haloketone (**2a**) in the presence of isopropanol upon prolonged irradiation time.

## Substrate Scope

The Cu–C_3_N_4_ displays high performance in the photosynthesis of a series of α-haloketones from the corresponding substrates under ambient conditions (Table 2). We have first attempted the synthesis of α-chloroketones that are important precursors for pharmaceutical applications^4,36^ but kinetically challenging due to the low activity of chlorine radicals. For the synthesis of α-chloroketones (**2a**–**2n**), substrates with electron-withdrawing groups (EWG, **2b**–**2f**) show in general high yields toward the target products, as the EWG will stabilize the α-carbon radicals to facilitate the attack of photogenerated oxygen radicals. In comparison, though the presence of electron-donating groups (EDG, **2g**–**2i**) is unfavorable for the formation of α-carbon radicals, the target products can still be obtained with reasonable selectivities (∼60%). Furthermore, substitution of the −Cl on the *ortho-* (**2j**) and *meta-* (**2k**) positions does not have a significant deleterious impact on the selectivity (>70%). Pleasingly, styrene substituted with a vulnerable ester group (**2l**) attached on the aromatic ring can also be converted into the corresponding α-chloroketone with a decent selectivity. Furthermore, the successful synthesis of 2-vinylnaphthalene (**2m**) suggests that the steric effect barely influences such a photocatalytic process. The synthesis of **2l** and **2m** sheds light on the synthesis of more complicated α-chloroketones for pharmaceutical applications.^37−40^ However, it is noticed that the conversion of α-methylstyrene into 2-chloropropiophenone (**2n**) shows a reduced selectivity, indicating that the α-methyl group strongly influences the electronic properties of the α-carbon-centered radicals. Additionally, a series of α-bromoketones (**3a**–**3n**) have also been synthesized by employing NiBr_2_ as the Br source. The impact of functional groups displays a similar trend observed in the synthesis of α-chloroketones, confirming a similar reaction mechanism. All bromination reactions complete in a much shorter reaction time (2 h) due to the higher activity of the bromine radicals.^5^

**Table 2:**
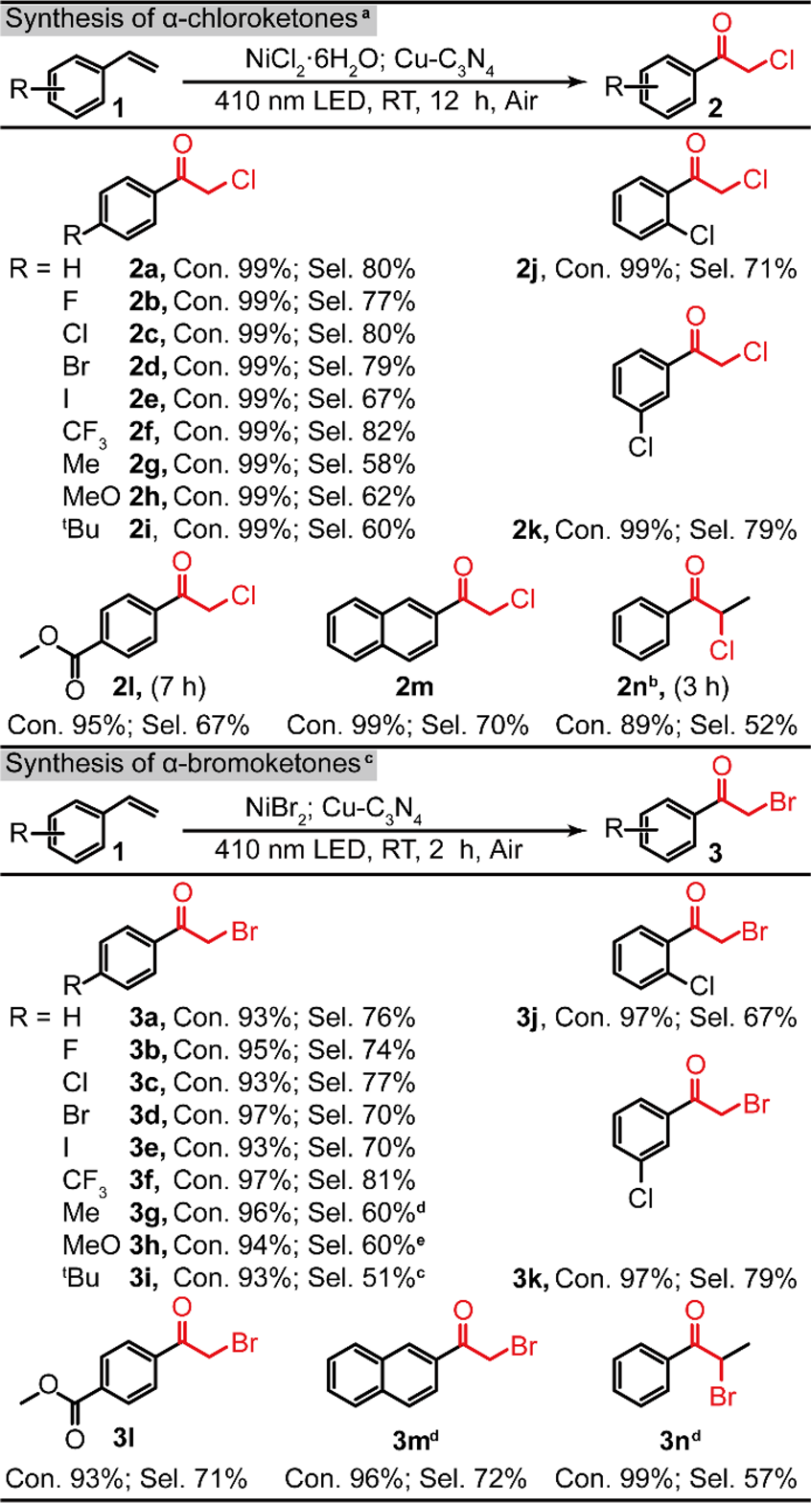
Substrate Scope: Synthesis of α-Haloketones Catalyzed by Cu–C_3_N_4_ under Ambient Conditions^f^

a 1 mL of isopropanol in 9 mL of ethyl acetate.

b 1 mL of ethanol in 9 mL of ethyl acetate.

c 0.1 mL of isopropanol in 1.9 mL of ethyl acetate.

d 0.1 mL of ethanol in 1.9 mL of ethyl acetate.

e 0.1 mL of isopropanol in 1.9 mL of 1,4-dioxane.

f Reaction conditions: The conversion and selectivity are determined by GC-MS.

In summary, we have designed a facile heterogeneous photocatalytic system for the synthesis of α-haloketones from aromatic olefins under visible-light irradiation. By employing Cu–C_3_N_4_ as the photocatalyst and NiX_2_ (X = Cl, Br) as the halogen source, a series of α-haloketones can be synthesized using atmosphere air as the oxidant. Mechanistic analysis reveals that the presence of Cu NPs as cocatalysts can optimize the formation kinetics of halogen radicals and the selective reduction of O_2_ into ^•^OOH radicals and promotes the desorption of generated H_2_O molecules, thus avoiding the formation of unwanted byproducts. Additionally, its high stability, versatility in expanding substrates, and capability for solar-driven photosynthesis reflect great potential for wider synthetic applications.

## Supporting Information Available

Experimental details, material characterizations, and NMR data (PDF)

## Notes

Information on the data underpinning the results presented here, including how to access them, can be found in the Cardiff University data catalogue at http://doi.org/10.17035/d.2022.0234944684
