# Supplementary material for: Copper Cocatalyst Modulated Radical Generation for Selective Heterogeneous Photosynthesis of α-Haloketones
Source: ACS Catal. 2022 Dec 15;13(1):248–55. doi: 10.1021/acscatal.2c05189 (PMC9830627; doi:10.1021/acscatal.2c05189)
Supplement: Supplementary file 1 — cs2c05189_si_001.pdf [file cs2c05189_si_001.pdf]

Supporting Information

Copper Cocatalyst Modulated Radical Generation for Selective Heterogeneous Photosynthesis of α-Haloketones

Feiyu Han,[a,b,#] Dongsheng Zhang,[a,b,#] Sofia Salli,[c,#] Jiani Ye,[a] Yongwang Li,[b,d] Federico Rosei,[e] Xiao-Dong Wen,[b,d] Hans Niemantsverdriet,[b,f] Emma Richards,[c,*] and Ren Su[a,b,*]

[a] Soochow Institute for Energy and Materials InnovationS (SIEMIS), Soochow University, 215006, China

[b] SynCat@Beijing, Synfuels China Technology Co. Ltd., Leyuan South Street II, No.1, Huairou, Beijing, 101407, China

[c] School of Chemistry, Cardiff University, Park Place, Cardiff, CF10 3AT, UK

[d] State Key Laboratory of Coal Conversion, Institute of Coal Chemistry, CAS, Taiyuan, 030001, China

[e] Center for Energy, Materials and Telecommunications, Institut National de la Recherche Scientifique, 1650 Boulevard Lionel-Boulet, Varennes, J3X 1P7 Québec, Canada

[f] SynCat@DIFFER, Syngaschem BV, 6336 HH Eindhoven, The Netherlands

[#] Contribute equally to this work

[*]Corresponding Author: Emma Richards (richardse10@cardiff.ac.uk); Ren Su (suren@suda.edu.cn).

Table of Contents

Experimental Procedures

Supporting Figures

Figure S1. Image of the leak tight reactor for the synthesis of Cu-C_3N_4.

Figure S2. XPS analysis of pristine g-C_3N_4 and Cu-C_3N_4.

Figure S3. Characterizations of the photocatalysts.

Figure S4. Byproducts analysis by GC-MS.

Figure S5. Photocatalytic synthesis of α-haloketones using ethanol and deuterated ethanol (C_2H_5OD) as hydrogen donor.

Figure S6. Photocatalytic performance at high concentrations of styrene.

Figure S7. ESR spectra under dark conditions.

Figure S8. Colorimetric titration of H_2O_2 formation using g-C_3N_4 and Cu-C_3N_4.

Figure S9. Image of the *in-situ* oxygen consumption analyzing system.

Figure S10. Reaction kinetic analysis of g-C_3N_4 and Cu-C_3N_4.

Figure S11-S38. Product analysis of photocatalytic synthesis of a series of α-haloketones using Cu-C_3N_4 photocatalyst by NMR.

Supporting Tables

Table S1. ICP-AES analysis of Cu loadings on Cu-C_3N_4.

Table S2. Control experiments of photocatalytic conversion of styrene.

Supporting References

Experimental Procedures

1. Synthesis of photocatalyst

Materials: Urea (Tianjin Beichen District Fangzheng Reagent Factory), $CuCl_2$ (Shanghai Aladdin Reagent Co. Ltd), isopropanol (99.0%, analytic grade, Shanghai Aladdin Bio-Chem Technology Co., Ltd.). All reagents were used directly without further purification unless otherwise stated.

1.1 Synthesis of g-C_3N_4

The g-C_3N_4 was prepared by a common pyrolysis method using urea as the precursor[1-2]. The urea powders were added into a 30 mL crucible with a lid, and heated at 550 °C for 3 h in air in a muffle oven with a ramping rate of 5 $^{\circ}C\cdot min^{-1}$.

1.2 Synthesis of Cu-C_3N_4

The Cu-C_3N_4 was prepared by a typical photo-deposition method[3]. First, g-C_3N_4 powders (0.5 g) were dispersed in 15 mL isopropanol at room temperature (RT) and subjected to ultrasonic treatment for 10 min. Then, 10.6 mg anhydrous $CuCl_2$ was dissolved in 10 mL DI water. The two solutions were mixed in a leak-tight glass reactor under magnetic stirring for 2 h. The resulting suspension was purged with N_2 gas 3 times to remove residual air. The suspension was irradiated using a 410 nm LED light for 2 h at RT under an inert atmosphere to reduce Cu cations into metallic Cu loaded on the g-C_3N_4. Finally, the suspension was filtered and washed three times with DI water, and dried at 60 °C in a vacuum drying oven for 12 h to obtain the Cu-C_3N_4. The image of leak tight reactor is shown in Figure S1.

2. Characterization of the catalysts

2.1 Spherical-aberration-corrected transmission electron microscopy (Cs-TEM)

The microscopic morphology of the photocatalyst was characterized using an FEI Titan Themis Cubed G2 300 microscope. The Cu-C_3N_4 powder samples were dispersed into ethanol and dropped on the Mo grid for analysis.

2.2 X-ray photoelectron spectroscopy (XPS)

The composition and valence of the elements on the surface of the catalyst material were characterized using an X-ray photoelectron spectrometer (XPS, K-Alpha, Thermo Fisher Scientific). The survey scans were recorded with the energy scan range from 1200 to -10 eV, using 1 eV step size and 140 eV pass energy, with a dwell time of 0.1 s. The region-of-interest spectra were measured with a step size and a pass energy of 0.1 eV and 26 eV, respectively, with a dwell time of 0.5 s. The peak of adventitious carbon (284.6 eV) was used for calibration. The results are presented in Figure S2.

2.3 Power X-ray diffraction (PXRD)

Diffraction patterns of the pristine g-C_3N_4 and Cu-C_3N_4 were measured using a Bruker D8 Advance diffractometer with a step size of 0.02 ° and a scan range of 5 ° to 60 ° using a Cu- Kα radiation source (40 kV, 40 mA). The results are shown in Figure S3a.

2.4 Diffuse Reflectance Spectroscopy (DRS)

A Hitachi photo-spectrometer (UH4150) equipped with a spherical integrating detector was employed to record the DRS profiles in the wavelength range of 300 to 800 nm. Spectroscopy grade $BaSO_4$ was used as the reference. The results and tauc plots of g-C_3N_4 and Cu-C_3N_4 are shown in Figures S3b and S3c.

2.5 Fourier transform infrared spectrometry (FTIR)

An infrared spectrometer (VERTEX 70, Bruker) was used to record diffuse reflectance infrared spectroscopy (Drift) of the photocatalysts using KBr as the reference. The results are shown in Figure S3d.

2.6 Photoluminescence spectroscopy (PL)

Photoluminescence spectra of g-C_3N_4 and Cu-C_3N_4 were measured by an Edinburgh fluorescence photometer (FLS1000). A xenon lamp with wavelength of 330 nm and 375 nm were used as light sources for steady state and time-resolved PL, respectively.

2.7 Inductively coupled plasma-atomic emission spectrometry (ICP-AES)

The ICP-AES was employed to quantify the loading of Cu on the photocatalysts. The result is shown in Table S1.

3. Photocatalytic performance evaluation

3.1 Supplementary experimental conditions for photocatalytic experiments

The conversion and selectivity were determined by gas chromatograph (GC) and gas chromatograph mass spectrometry (GC-MS) using commercial standard compounds (styrene and 2-chloroacetophenone) at different concentrations (2 mM, 4 mM, 8 mM, 10 mM, 25 mM, 50 mM). The chemical structures of the products were confirmed by the GC-MS, ^{1}H NMR and ^{13}C NMR spectra.

A series of control experiments are shown in Table S2. A combination of Ni-C_3N_4 and $CuCl_2$ shows poor conversion of styrene into the α-haloketone (Entry 1), indicating the irreplaceable role of copper metal on the catalyst surface. There is no formation of product without either the Cu-

C_3N_4 photocatalyst, a suitable chlorine source, light or presence of atmospheric oxygen (Entries 2-5).

3.2 Solar photocatalytic synthesis of α-haloketone

The solar photocatalytic experiment was performed using a 25 mL round-bottom flask with a rubber sealing plug under magnetic stirring. The reactor was placed on an open roof, and the solar intensity was recorded every hour. Reaction conditions: 75 mg photocatalyst (Cu-C_3N_4), 0.3 mmol halogen source ($NiCl_2 \cdot 6H_2O$), solvent (1.5 mL isopropanol in 13.5 mL ethyl acetate), 8 mM reactant.

3.3 Photosynthesis of α-haloketones using deuterated hydrogen donor

The synthesis of α-haloketones was performed using deuterium ethanol (C_2H_5OD) instead of isopropanol but otherwise identical reaction conditions. The MS spectrum of the product is shown in Fig S5 in comparison with α-haloketones synthesized using ethanol (C_2H_5OH).

3.4 Photocatalytic performance at high concentrations of styrene

Photosynthesis of α-haloketones was performed at different concentrations of styrene for irradiation times under standard reaction conditions (12 h for 8 and 16 mM, 16 h for 24 mM and 20 h for 32 mM). The performance is shown in Fig S6.

4. Mechanism analysis

4.1 Electron Spin Resonance (ESR)

The radicals generated during the photocatalytic reaction process were analyzed by electron spin resonance (ESR) using an X-band JES-X320 spectrometer in the range of 321-331 mT at RT. An receiver gain of 400 and a modulation width of 0.05 mT (modulation frequency = 100 kHz) was

used for all measurements. The specific steps and details of the ESR experiments are described as the following:

For oxygen radicals: A 10 mg photocatalyst powder was dispersed in 1 mL of 1,4-dioxane with stirring overnight. Prior to analysis, the spin-trap solution (20 mM of 5,5-dimethyl-1-pyrroline N-oxide, DMPO) was dosed into the suspension and exposed to blue light irradiation (410 nm) at RT for 1.5 min.

For chlorine radicals: A 10 mg photocatalyst powder and 0.02 mmol halogen source ($NiCl_2 \cdot 6H_2O$, NaCl, $FeCl_3$ or $CuCl_2$) were dispersed in 1 mL of 1,4-dioxane with stirring overnight. Prior to analysis, the spin-trap solution (20 mM of N-tert-butyl-α-phenylnitrone, PBN) was dosed into the suspension and exposed to blue light irradiation (410 nm) at room temperature for 1.5 min.

The ESR spectra recorded under dark are shown in Figure S7.

4.2 Colorimetric titration of H_2O_2 evolution under irradiation

The generation of H_2O_2 is quantitatively analyzed by UV-vis spectrometry (UH4150 spectrophotometer, Hitachi). The photogenerated H_2O_2 was gauged by a colorimetric titration method using a Cu^{2+} based chromogenic agent[4-5]. The chromogenic agent is prepared by mixing 2,9-dimethyl-1,10-phenanthroline (DMP) ethanol solution (1 $mg \cdot mL^{-1}$) and aqueous $CuSO_4$ solution (0.01 M) in 1:1 volume ratio.

For the photocatalytic generation of H_2O_2, 50 mg photocatalyst powders were added into 10 mL solvent (1 mL isopropanol in 9 mL ethyl acetate) under continuous stirring in air at RT for irradiation. The suspension was centrifuged at desired irradiation intervals and 200 μL of aliquots was added into 2.8 mL of the chromogenic agent. The resulting solution was kept for 1 minute prior to UV-vis spectroscopy analysis.

According to the previous work[6], one part of H_2O_2 can oxidize two part of Cu(II)-DMP complex into Cu (I)-DMP complex. We have prepared a series of H_2O_2 standard solution to determine the absorption coefficient (ε) of the Cu(I)-DMP complex (Figure S8a), which is 2.33×10^4 $M^{-1}\cdot cm^{-1}$ according to the linear fit (Figure S8b). The photographs of the colorimetric titration of H_2O_2 using g-C_3N_4 and Cu-C_3N_4 photocatalysts are shown in Figure S8c and S8d.

4.3 In-situ analysis of oxygen consumption

The oxygen consumption during the reaction process was probed by an *in-situ* leak-tight oxygen concentration analyzing system that is equipped with a TO2-1x PPM oxygen sensor, Southland Sensing, as shown in Figure S9. For the photocatalytic consumption of O_2, 50 mg photocatalyst powders and 0.2 mmol $NiCl_2\cdot6H_2O$ were added into 10 mL solvent (1 mL isopropanol in 9 mL ethyl acetate) under continuous stirring in air at RT. The mixed reaction suspension was then transferred to the reactor in the glove box. Prior to irradiation, the reactor was purged with a 500 ppm O_2/N_2 standard gas for 10 min and sealed till the O_2 concentration reached a stable reading. The 410 nm LED light was turned on for 1 h (30 $m\cdot Wcm^{-2}$), and the oxygen concentration was measured continuously. Lastly, the oxygen concentration was measured for an additional 10 min after switching off the irradiation.

4.4 Temperature programmed desorption of water (H_2O-TPD)

The H_2O-TPD of g-C_3N_4 and Cu-C_3N_4 were accomplished by using a chemisorption analyzer (AutoChem II) coupled with a MS (OmniStar GSD, Pfeiffer). The specific steps and details of the H_2O-TPD experiments are described as follows:

First, the photocatalyst powder samples were soaked with DI water and vacuum dried overnight in RT. Then a 20 mg of sample was transferred into a glass U-tube. Prior to temperature raising, the sample was purged by He flow for 0.5 h. The TPD profile of water ($m/e^- = 18$) was collected

from 40 to 300 ºC with a ramping rate of 10 ºC·min^{-1}. Helium was used as the carrier gas with a flow rate of 20 mL·min^{-1} in the whole process.

Supporting Figures

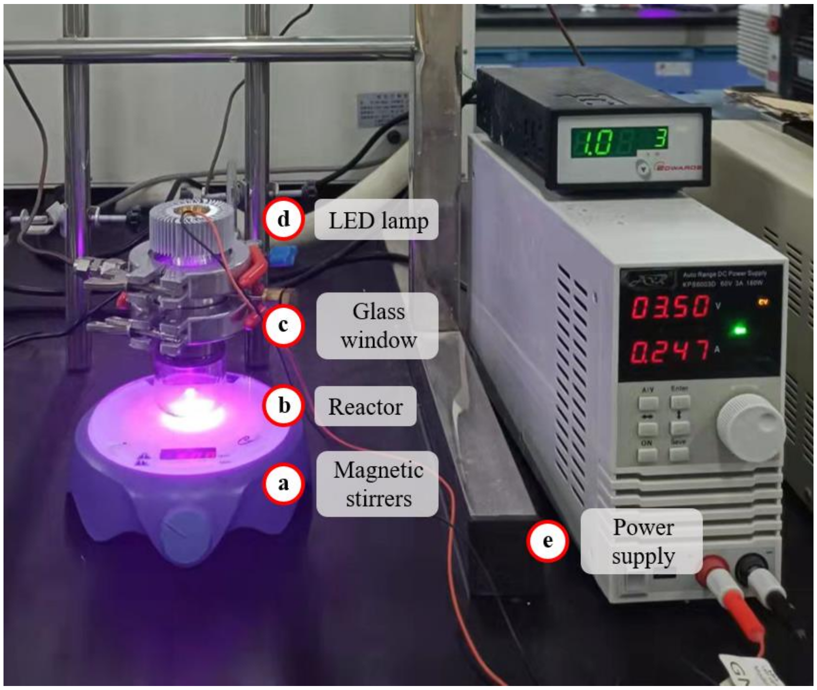

Figure S1. Image of the leak tight reactor for the synthesis of Cu-C_3N_4. The reactor (b) is placed on a magnetic stirrer (a). The LED light (d) is connected to a power supply (e) and placed on top of the quartz window (c).

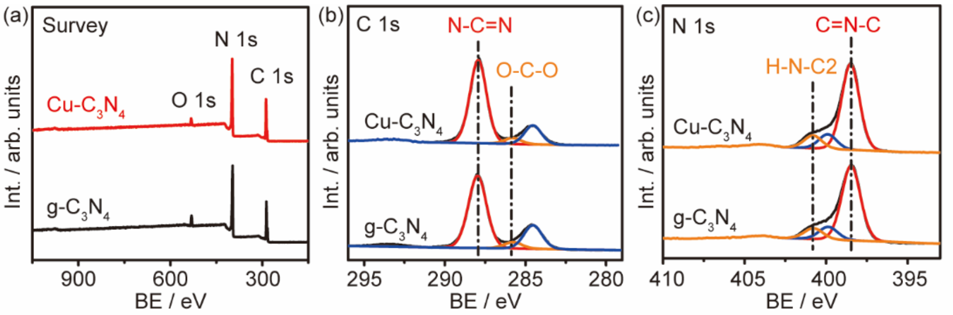

Figure S2. XPS analysis of pristine g-C_3N_4 and Cu-C_3N_4. (a) survey, (b) C1s and (c) N1s for pristine g-C_3N_4 and Cu-C_3N_4.

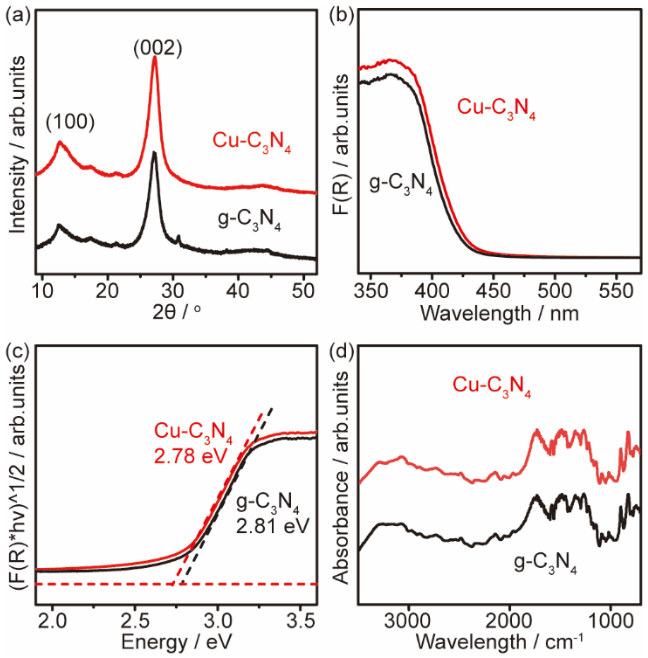

Figure S3. Characterizations of the photocatalysts. (a) XRD patterns of g-C_3N_4 and Cu-C_3N_4. (b) DRS and (c) Tauc plots of g-C_3N_4 and Cu-C_3N_4. (d) Drift of g-C_3N_4 and Cu-C_3N_4.

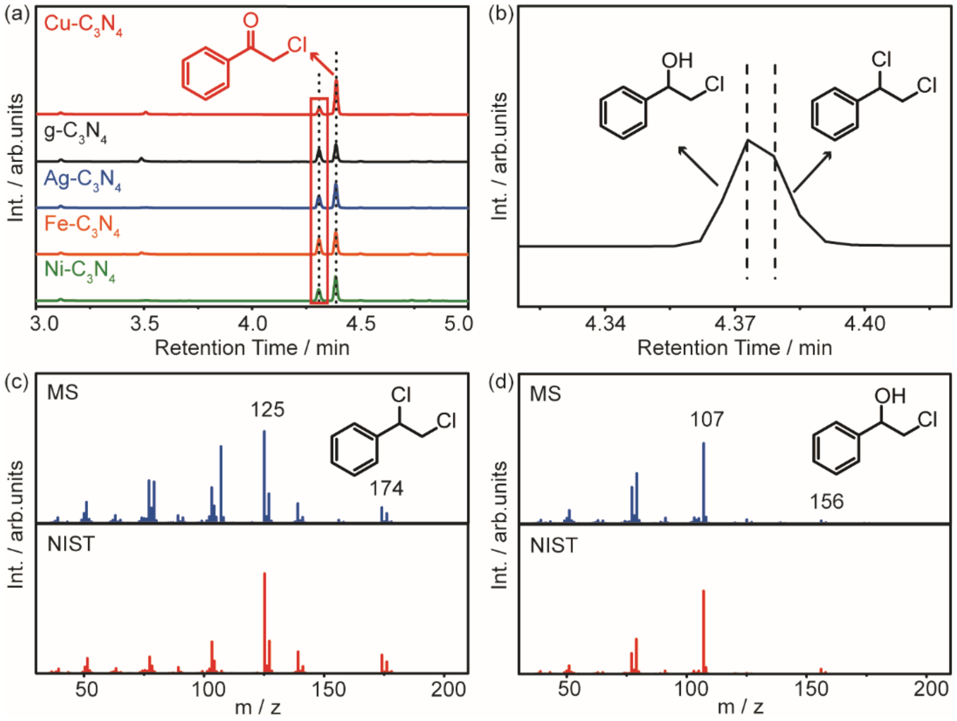

Figure S4. Byproducts analysis by GC-MS. (a) and (b) GC analysis of all products generated using different photocatalysts after irradiation. (b) is the zoom-in of the mark region in (a). (c) and (d) MS of the marked byproducts di-chloro-ethylbenzene and 2-chloro-1-phenylethanol (blue lines), respectively. The standard MS of the corresponding compounds from NIST database are also provided (red lines).

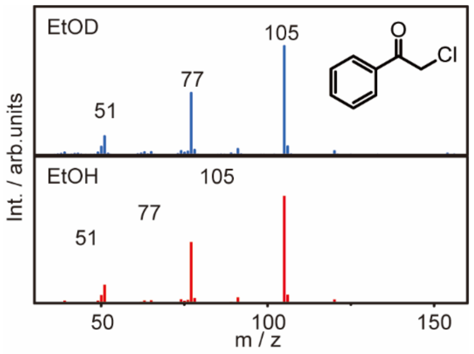

Figure S5. Photocatalytic synthesis of α-haloketones using ethanol and deuterated ethanol (C_2H_5OD) as hydrogen donor.

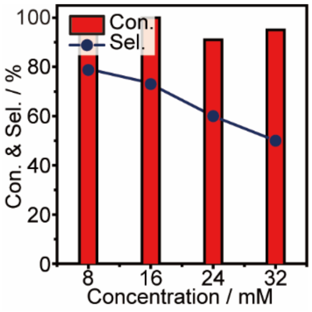

Figure S6. Photocatalytic performance at high concentrations of styrene.

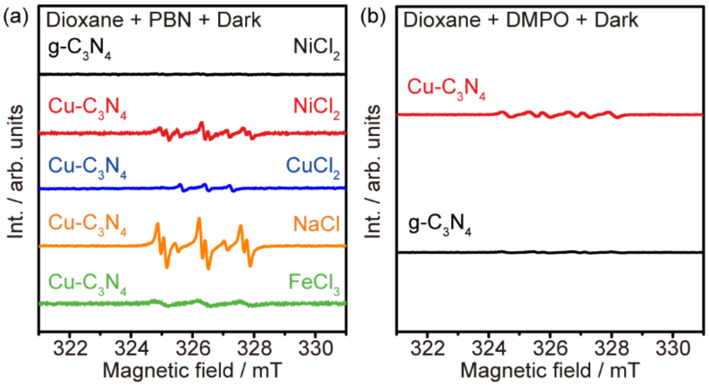

Figure S7. ESR spectra under dark conditions. (a) ESR spectra recorded in the presence of metal chlorides and PBN spin trap using Cu-C_3N_4 and g-C_3N_4 photocatalysts. (b) ESR spectra recorded in the presence of DMPO spin trap using Cu-C_3N_4 and g-C_3N_4 photocatalysts with the absence of metal chlorides.

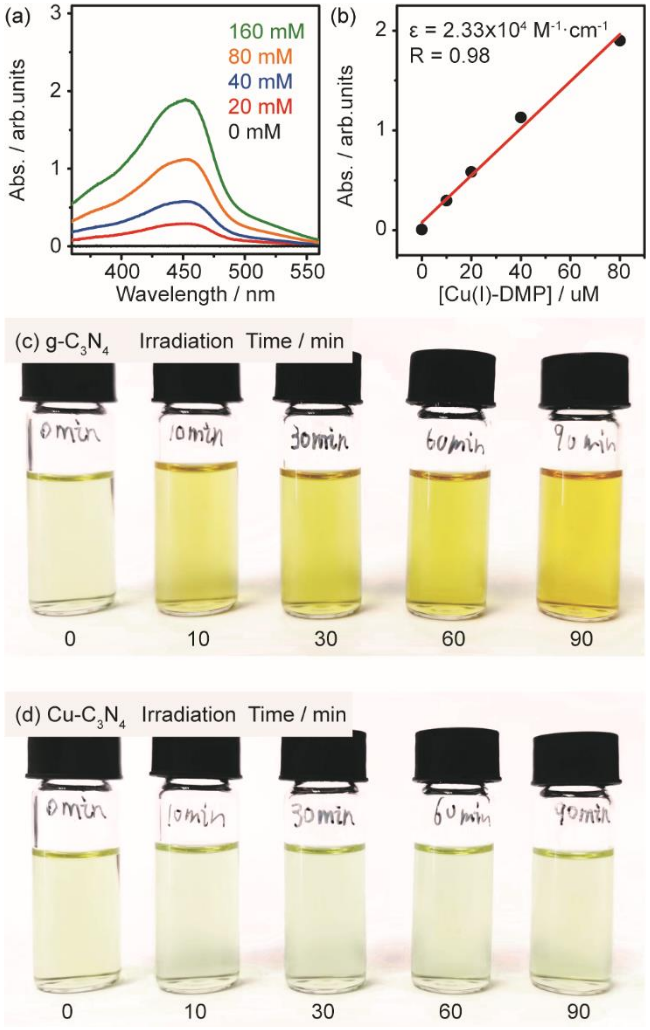

Figure S8. Colorimetric titration of H_2O_2 formation using g-C_3N_4 and Cu-C_3N_4. (a) UV-vis spectra of a series H_2O_2 standard solution titrated by the Cu(II)-DMP complex solution. The absorption peak of generated Cu(I)-DMP is located at 450 nm. (b) Determination of the absorption coefficient of derived Cu (I)-DMP complex. (c) and (d) Images of the colorimetric titration of H_2O_2 using g-C_3N_4 and Cu-C_3N_4 photocatalysts at different irradiation time, respectively.

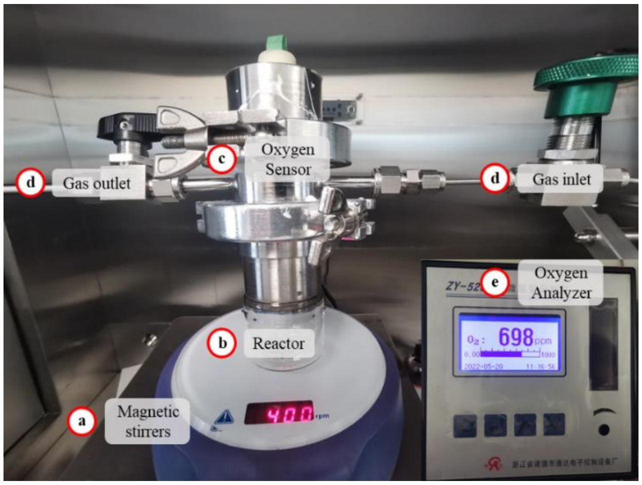

Figure S9. Image of the *in-situ* oxygen consumption analyzing system. The reactor (b) is placed on the magnetic stirrer (a) and combined with an oxygen sensor (c) and gas line (d). The oxygen analyzer (e) is connected with the oxygen sensor (c).

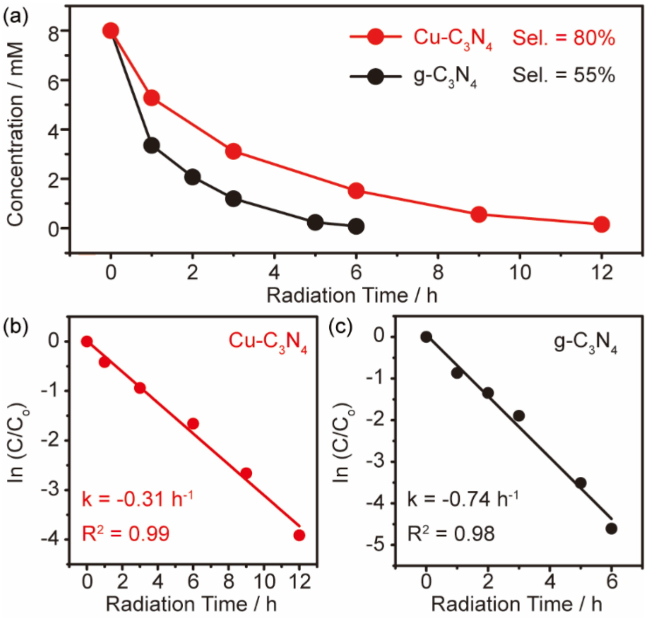

Figure S10. Reaction kinetic analysis of g-C_3N_4 and Cu-C_3N_4. (a) Time-course of photocatalytic styrene conversion using Cu-C_3N_4 and g-C_3N_4. Reaction conditions: 50 mg photocatalyst and 0.2 mmol $NiCl_2\cdot6H_2O$ in 10 mL solvent with 8 mM reactant under 410 nm irradiation (30 $mW\cdot cm^{-2}$) and 1 bar air at RT. (b) and (c) kinetic analysis of photocatalytic conversion of styrene using Cu-C_3N_4 and g-C_3N_4.

Detailed product analyses are shown in Supplementary Figures S11-S38.

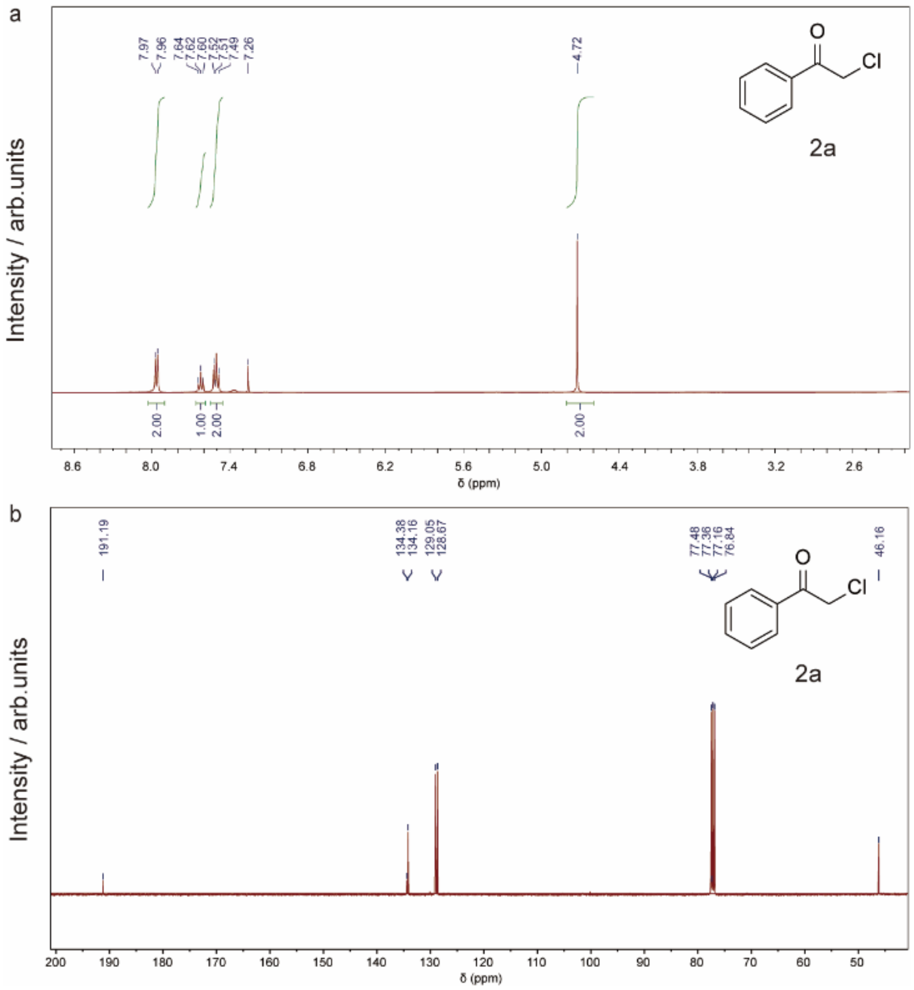

Figure S11. NMR spectra of 2a. (a) ^{1}H NMR (400 MHz, $CDCl_3$) δ 7.97 (d, J = 7.2 Hz, 2H), 7.62 (t, J = 8.0 Hz, 1H), 7.55 – 7.46 (m, 2H), 4.72 (s, 2H). (b) ^{13}C NMR (100 MHz, $CDCl_3$) δ 191.19, 134.38, 134.16, 129.05, 128.67, 46.16. The spectral data of this chemical matches previous work[7].

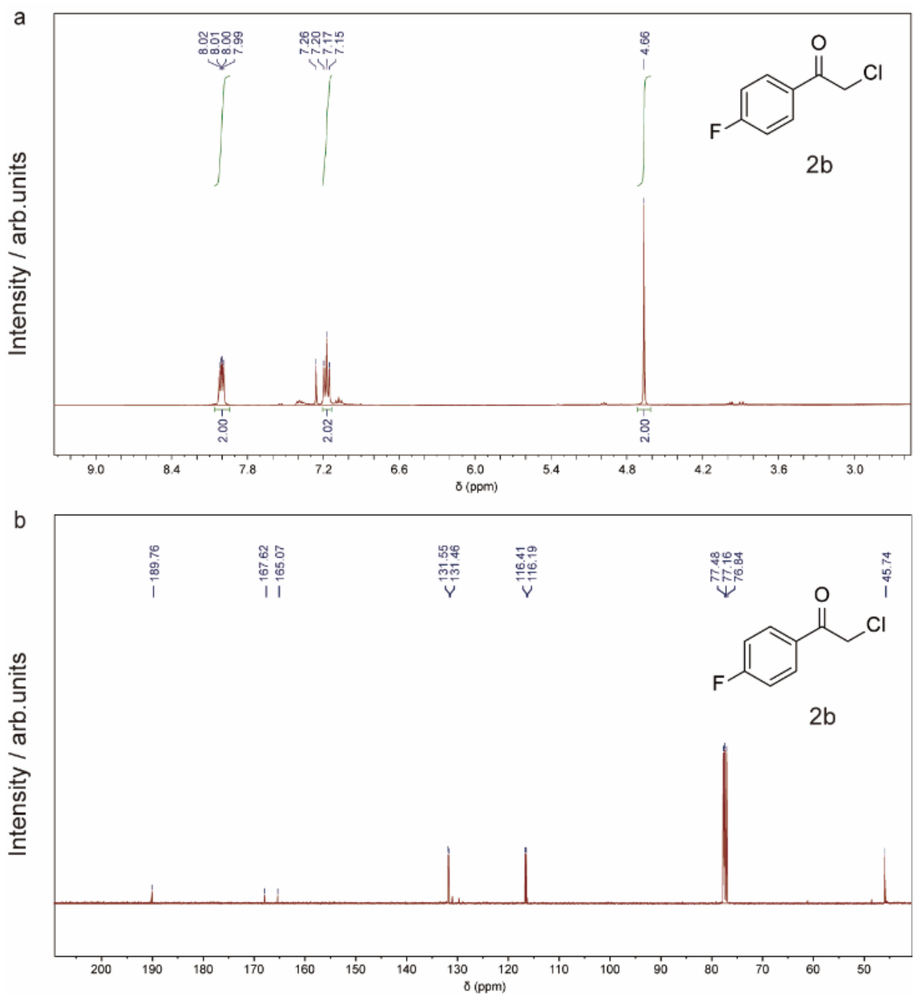

Figure S12. NMR spectra of 2b. (a) ^{1}H NMR (400 MHz, $CDCl_3$) δ 8.01 (dd, J = 8.8, 5.3 Hz, 2H), 7.17 (t, J = 8.6 Hz, 2H), 4.66 (s, 2H). (b) ^{13}C NMR (100 MHz, $CDCl_3$) δ 189.76, 167.62, 165.07, 131.55, 134.46, 116.41, 116.19, 45.74. The spectral data of this chemical matches previous work[7].

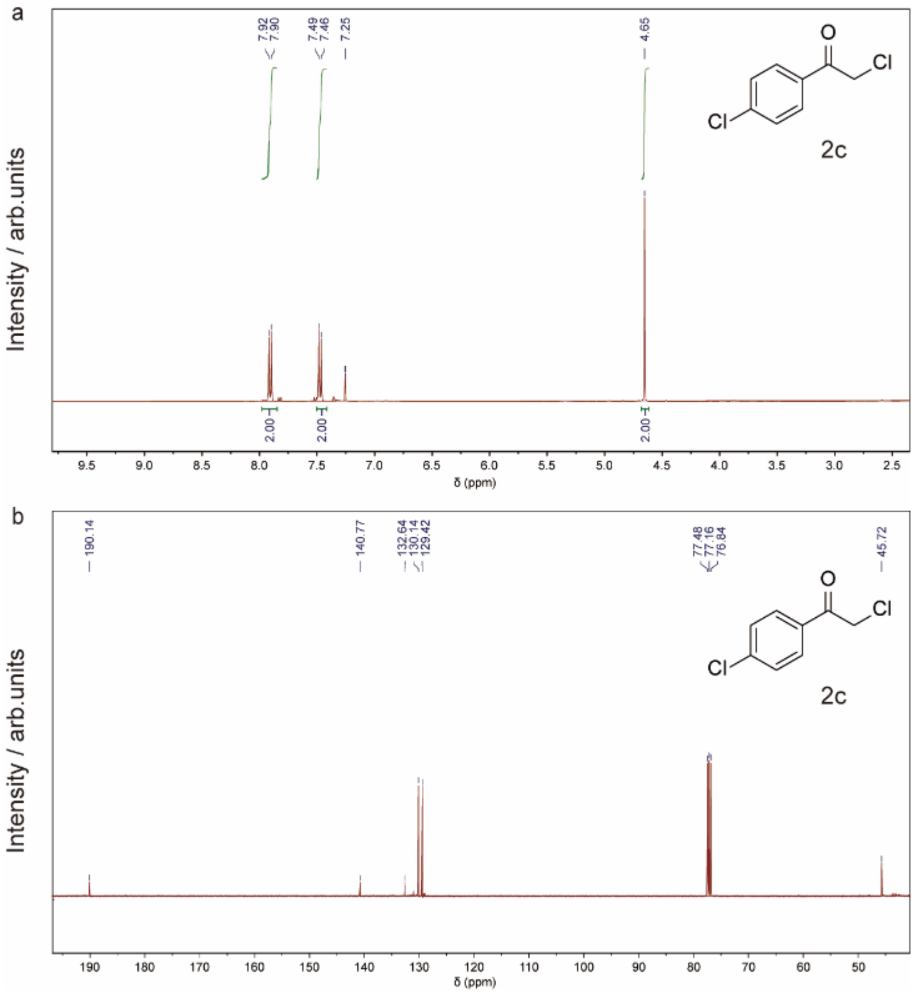

Figure S13. NMR spectra of 2c. (a) ^{1}H NMR (400 MHz, $CDCl_3$) δ 7.91 (d, J = 8.6 Hz, 2H), 7.47 (d, J = 8.6 Hz, 2H), 4.65 (s, 2H). (b) ^{13}C NMR (100 MHz, $CDCl_3$) δ 190.14, 140.77, 132.64, 130.14, 129.42, 45.72. The spectral data of this chemical matches previous work[7].

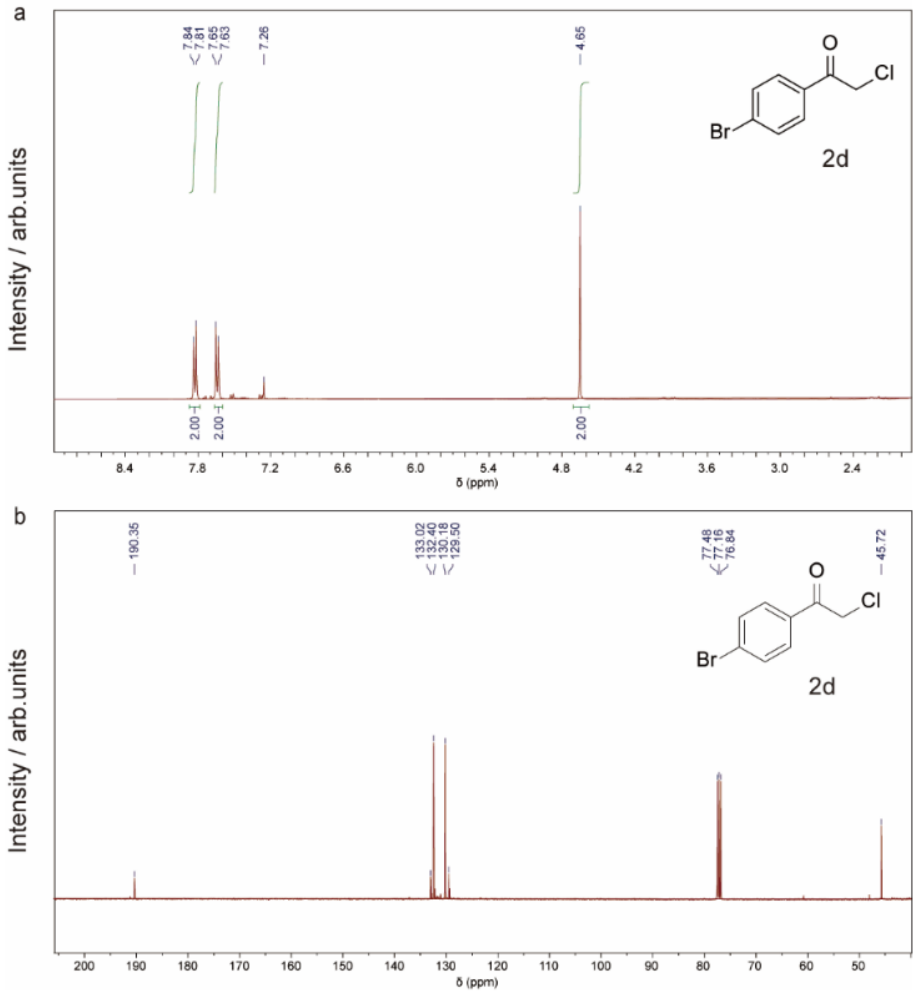

Figure S14. NMR spectra of 2d. (a) ^{1}H NMR (400 MHz, $CDCl_3$) δ 7.83 (d, J = 8.6 Hz, 2H), 7.64 (d, J = 8.6 Hz, 2H), 4.65 (s, 2H). (b) ^{13}C NMR (100 MHz, $CDCl_3$) δ 190.35, 133.02, 132.40, 130.18, 129.50, 45.72. The spectral data of this chemical matches previous work[7].

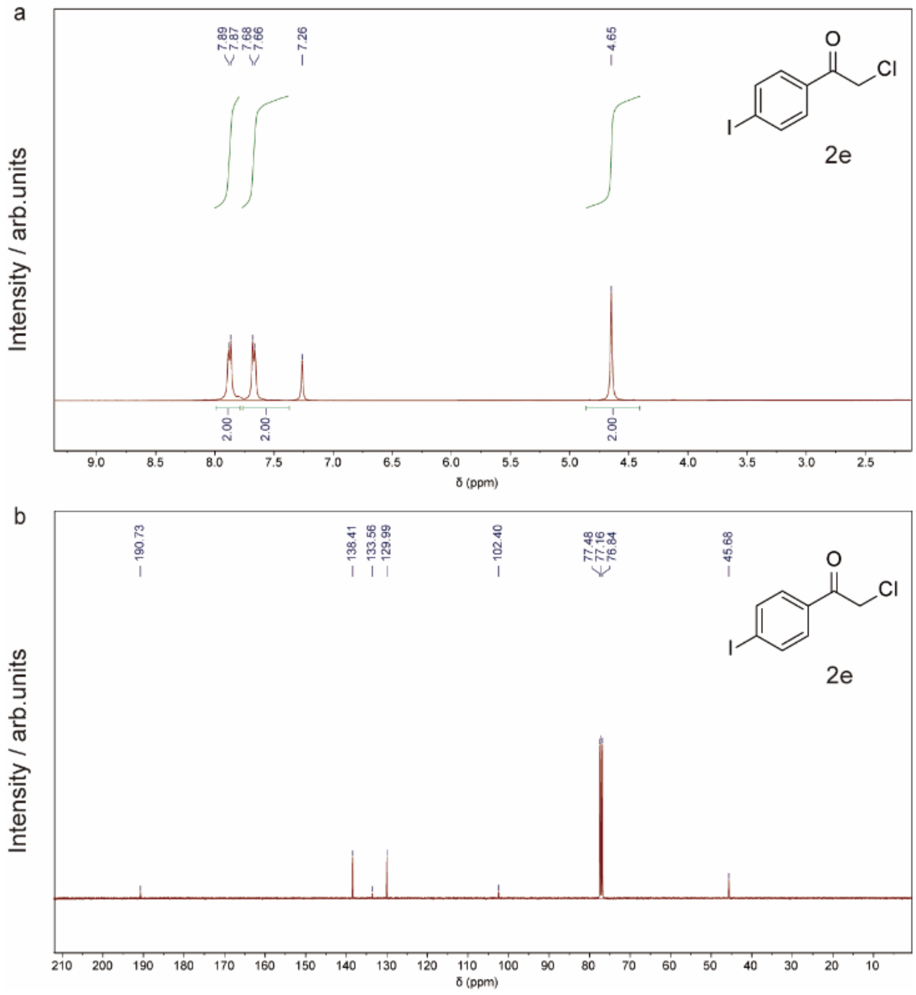

Figure S15. NMR spectra of 2e. (a) ^{1}H NMR (400 MHz, $CDCl_3$) δ 7.88 (d, J = 7.7 Hz, 2H), 7.67 (d, J = 7.6 Hz, 2H), 4.65 (s, 2H). (b) ^{13}C NMR (100 MHz, $CDCl_3$) δ 190.73, 138.41, 133.56, 129.99, 102.40, 45.68.

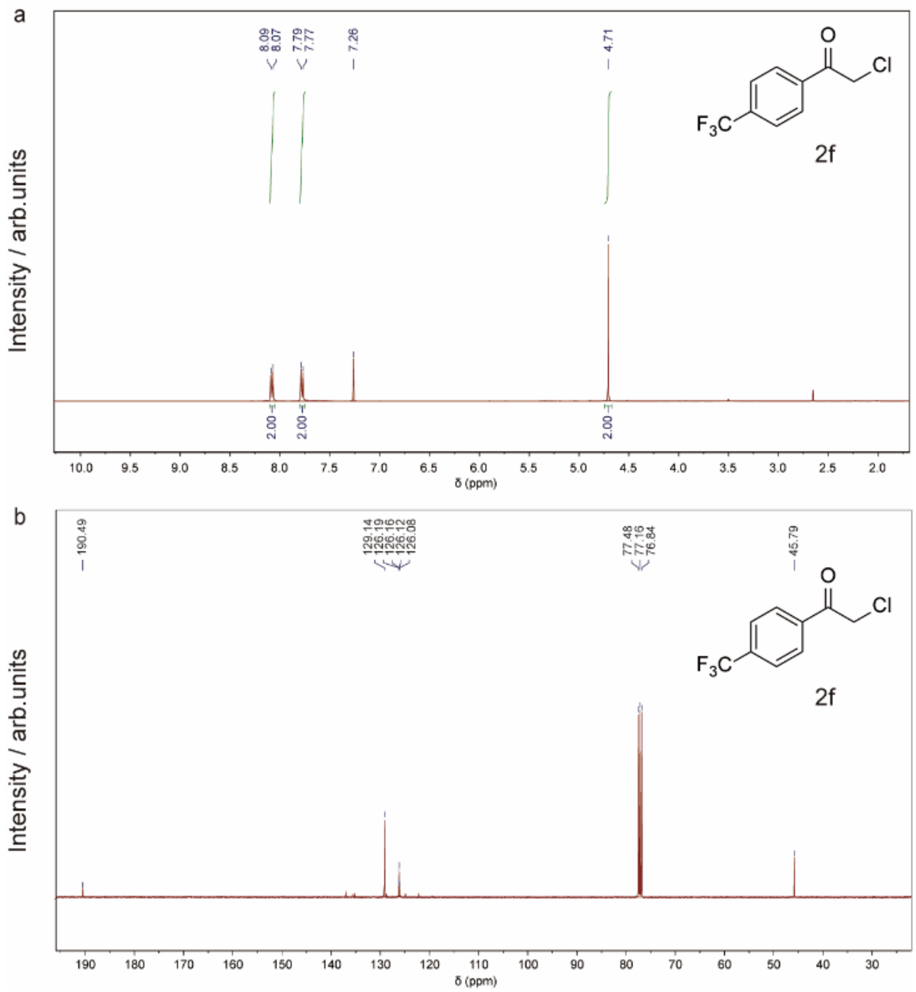

Figure S16. NMR spectra of 2f. (a) ^{1}H NMR (400 MHz, $CDCl_3$) δ 8.08 (d, J = 8.1 Hz, 2H), 7.78 (d, J = 8.2 Hz, 2H), 4.71 (s, 2H). (b) ^{13}C NMR (100 MHz, $CDCl_3$) δ 190.49, 129.14, 126.19, 126.16, 126.12, 126.08, 45.79. The spectral data of this chemical matches previous work[8].

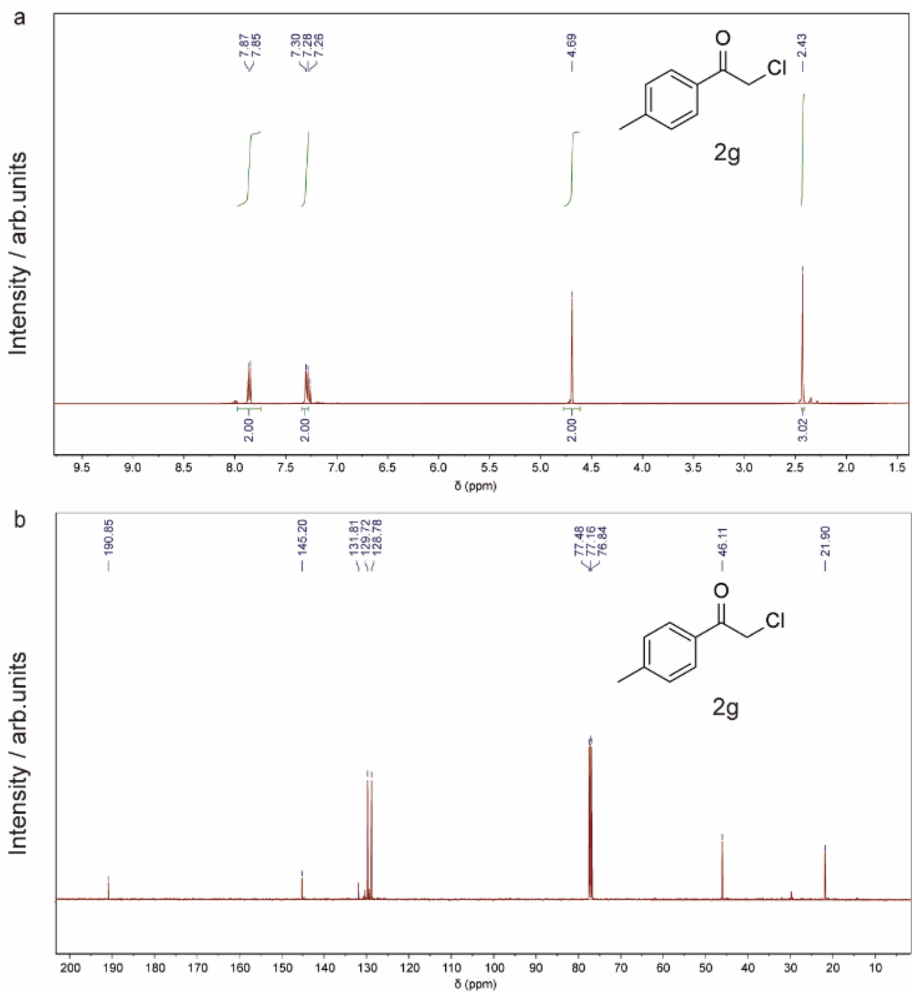

Figure S17. NMR spectra of 2g. (a) ^{1}H NMR (400 MHz, $CDCl_3$) δ 7.86 (d, J = 8.2 Hz, 2H), 7.29 (d, J = 8.1 Hz, 2H), 4.69 (s, 2H), 2.43 (s, 3H). (b) ^{13}C NMR (100 MHz, $CDCl_3$) δ 190.85, 145.20, 131.81, 129.72, 128.78, 46.11, 21.90. The spectral data of this chemical matches previous work[7].

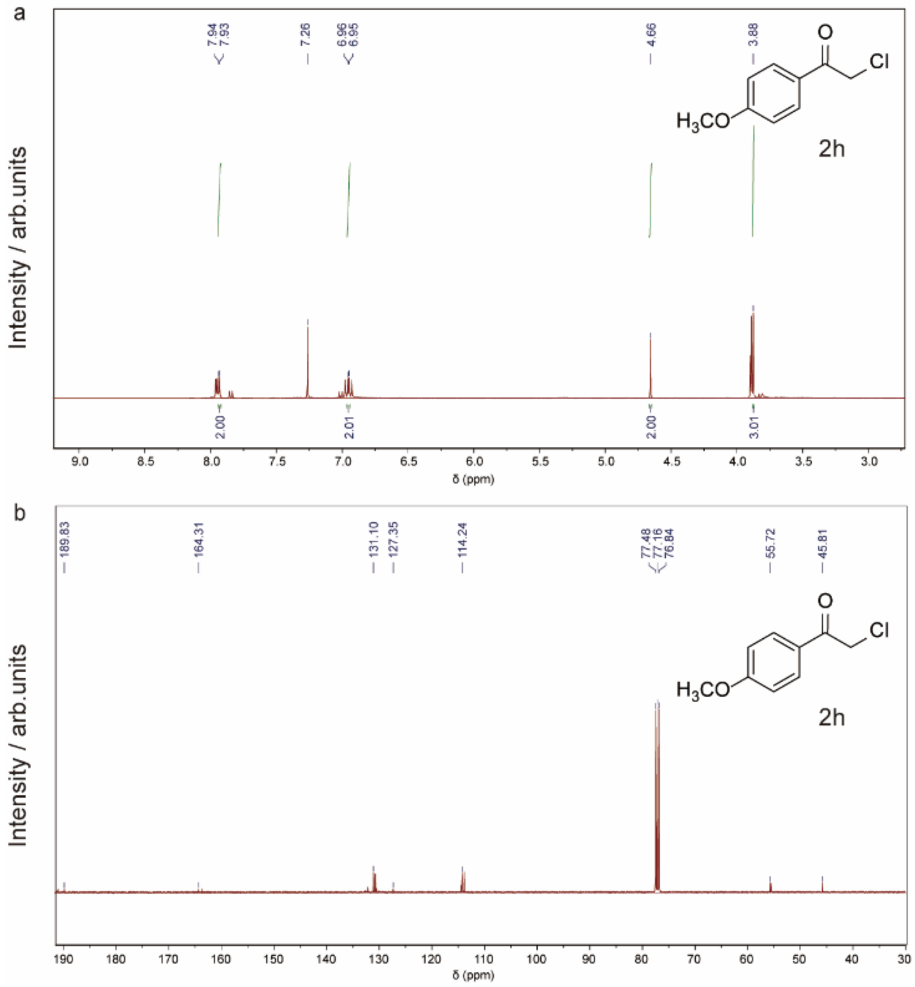

Figure S18. NMR spectra of 2h. (a) ^{1}H NMR (400 MHz, $CDCl_3$) δ 7.94 (d, J = 3.1 Hz, 2H), 6.95 (d, J = 2.9 Hz, 2H), 4.66 (s, 2H), 3.88 (s, 3H). (b) ^{13}C NMR (100 MHz, $CDCl_3$) δ 189.83, 164.31, 131.10, 127.35, 114.24, 55.72, 45.81. The spectral data of this chemical matches previous work[7].

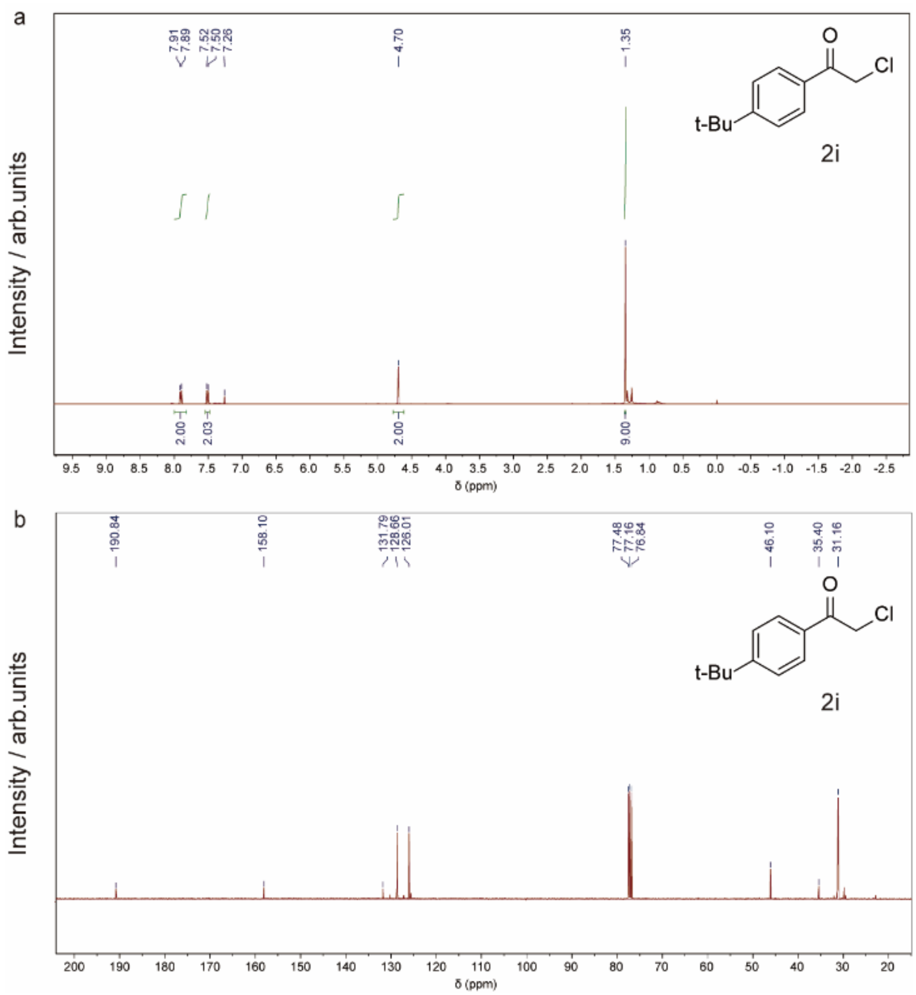

Figure S19. NMR spectra of 2i. (a) ^{1}H NMR (400 MHz, $CDCl_3$) δ 7.90 (d, J = 8.5 Hz, 2H), 7.51 (d, J = 8.5 Hz, 2H), 4.70 (s, 2H), 1.35 (s, 9H). (b) ^{13}C NMR (100 MHz, $CDCl_3$) δ 190.84, 158.10, 131.79, 128.66, 126.01, 46.10, 35.40, 31.16. The spectral data of this chemical matches previous work[8].

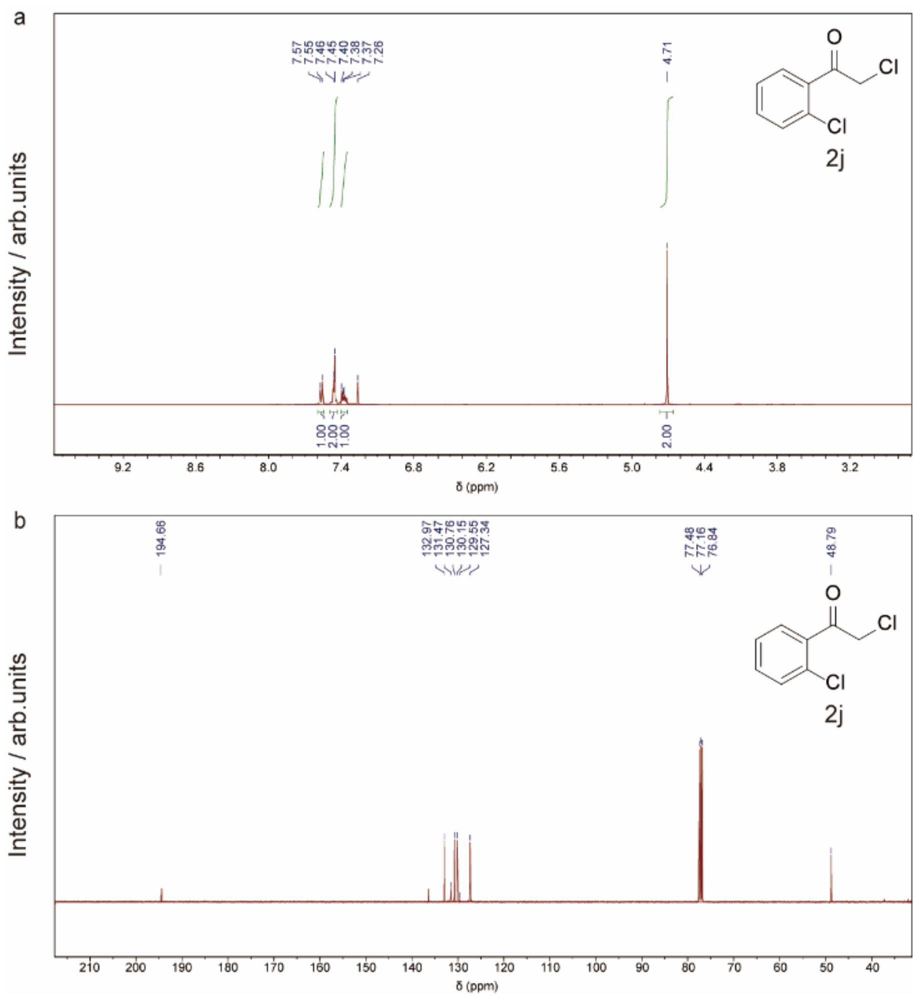

Figure S20. NMR spectra of 2j. (a) ^{1}H NMR (400 MHz, $CDCl_3$) δ 7.56 (d, J = 7.3 Hz, 1H), 7.46 (d, J = 2.5 Hz, 2H), 7.40 – 7.35 (m, 1H), 4.71 (s, 2H). (b) ^{13}C NMR (100 MHz, $CDCl_3$) δ 194.66, 132.97, 131.47, 130.76, 130.15, 129.55, 127.34, 48.79. The spectral data of this chemical matches previous work[7].

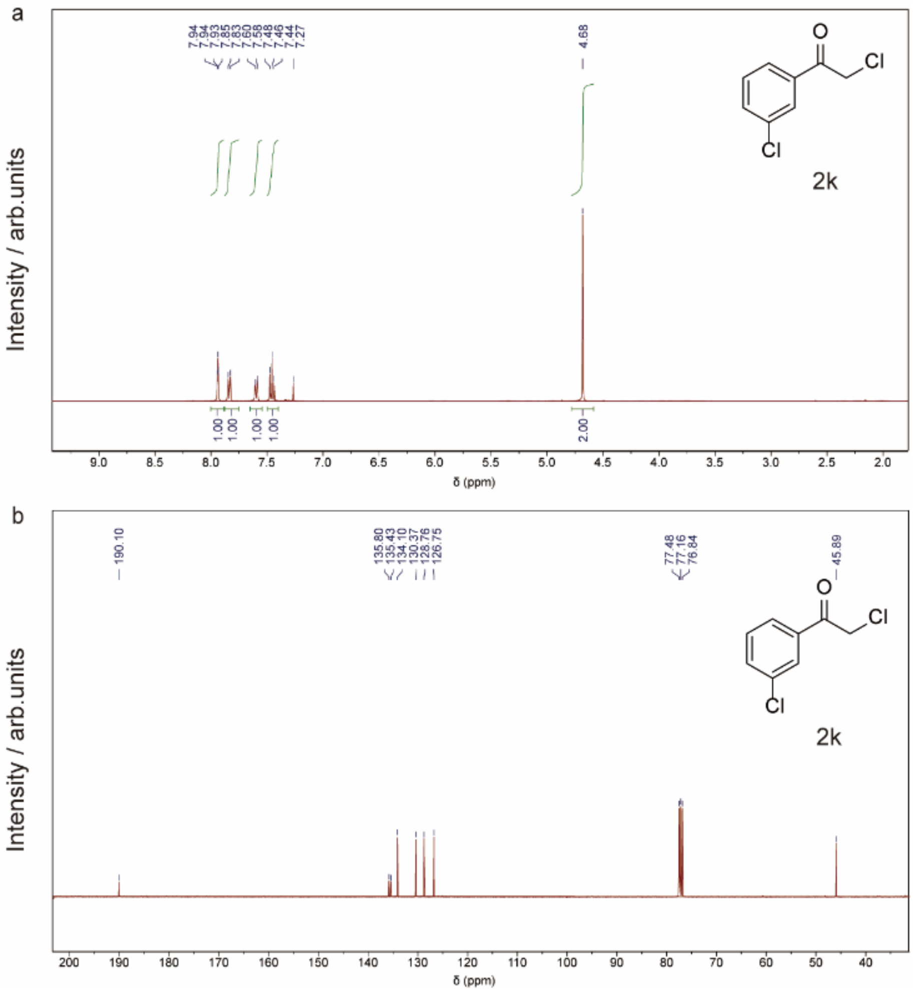

Figure S21. NMR spectra of 2k. (a) ^{1}H NMR (400 MHz, $CDCl_3$) δ 7.94 (t, J = 1.8 Hz, 1H), 7.84 (d, J = 7.8 Hz, 1H), 7.59 (d, J = 8.0 Hz, 1H), 7.46 (t, J = 7.9 Hz, 1H), 4.68 (s, 2H). (b) ^{13}C NMR (100 MHz, $CDCl_3$) δ 190.10, 135.80, 135.43, 134.10, 130.37, 128.76, 126.75, 45.89. The spectral data of this chemical matches previous work[8].

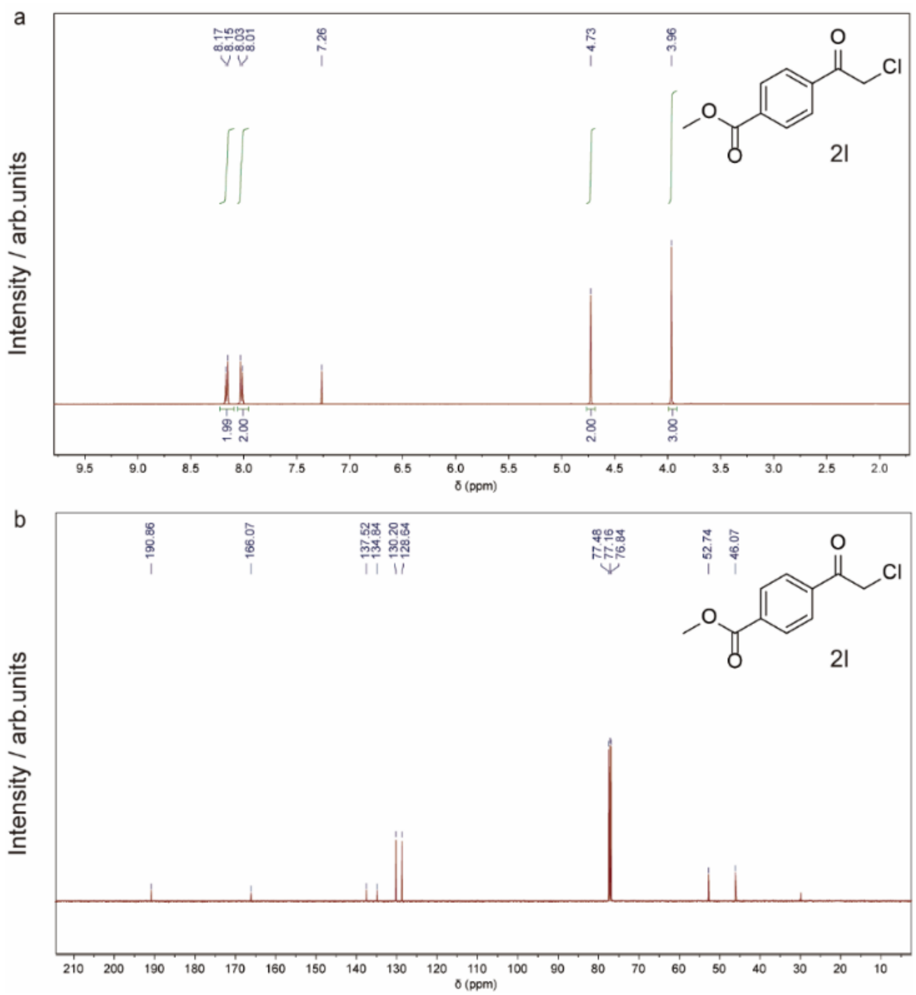

Figure S22. NMR spectra of 2l. (a) ^{1}H NMR (400 MHz, $CDCl_3$) δ 8.16 (d, J = 8.4 Hz, 2H), 8.02 (d, J = 8.4 Hz, 2H), 4.73 (s, 2H), 3.96 (s, 3H). (b) ^{13}C NMR (100 MHz, $CDCl_3$) δ 190.86, 186.07, 137.52, 134.84, 130.20, 128.64, 52.74, 46.07. The spectral data of this chemical matches previous work[8].

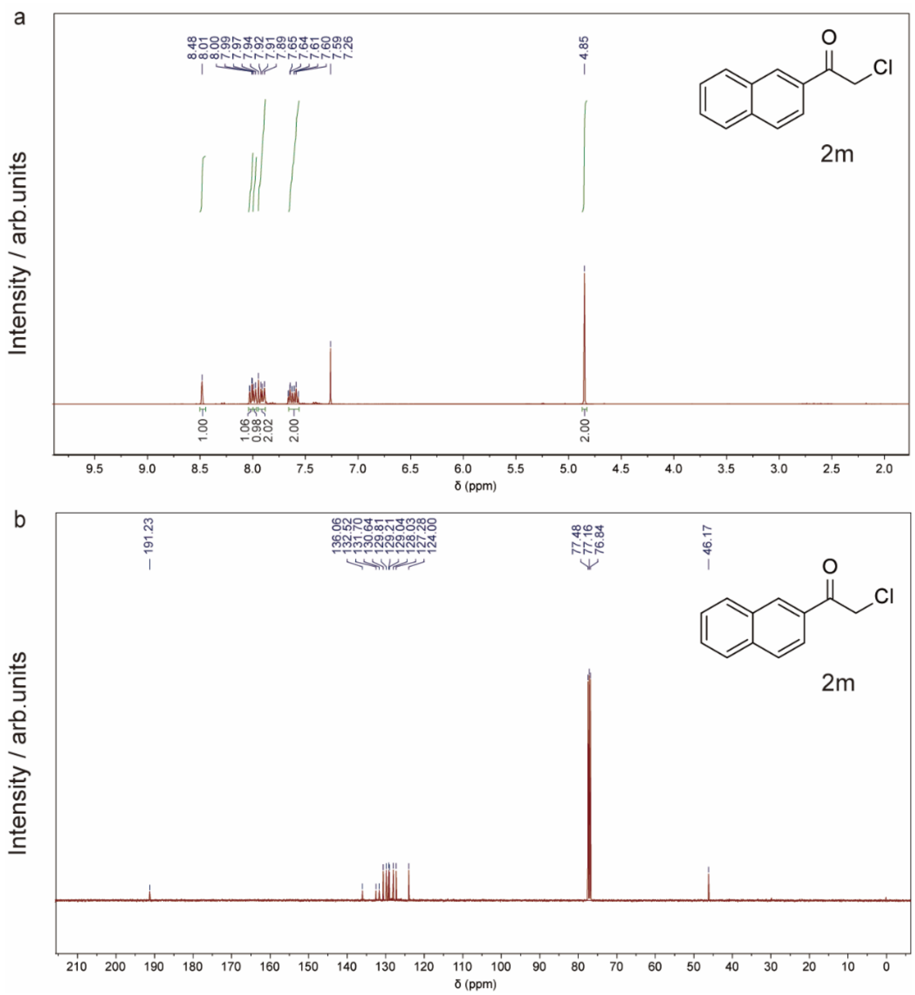

Figure S23. NMR spectra of 2m. (a) ^{1}H NMR (400 MHz, $CDCl_3$) δ 8.48 (s, 1H), 8.02 (dd, J = 8.6, 1.8 Hz, 1H), 7.98 (d, J = 8.1 Hz, 1H), 7.92 (dd, J = 14.2, 8.3 Hz, 2H), 7.67 – 7.55 (m, 2H), 4.85 (s, 2H). (b) ^{13}C NMR (100 MHz, $CDCl_3$) δ 191.23, 136.06, 132.52, 131.70, 130.64, 129.81, 129.21, 129.04, 128.03, 127.28, 124.00, 46.17. The spectral data of this chemical matches the previously work[8].

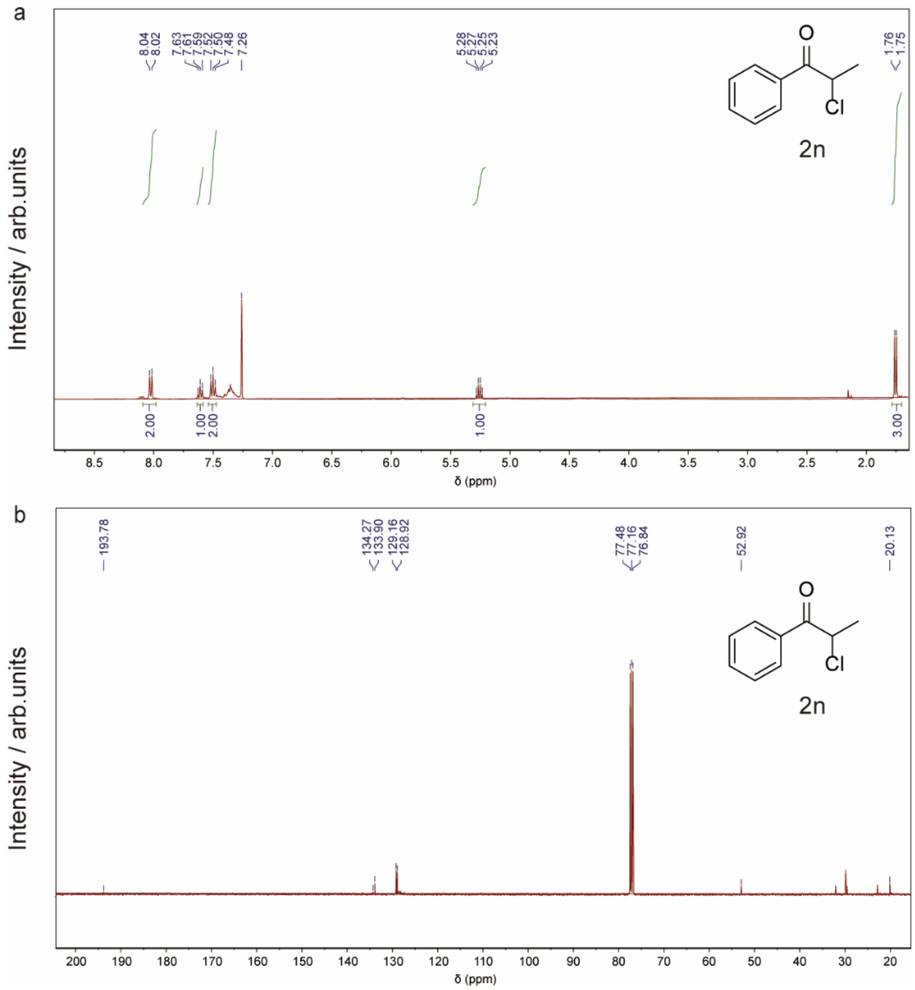

Figure S24. NMR spectra of 2n. (a) ^{1}H NMR (400 MHz, $CDCl_3$) δ 8.03 (d, J = 7.5 Hz, 2H), 7.61 (t, J = 7.4 Hz, 1H), 7.50 (t, J = 7.6 Hz, 2H), 5.26 (q, J = 6.6 Hz, 1H), 1.75 (d, J = 6.7 Hz, 3H). (b) ^{13}C NMR (100 MHz, $CDCl_3$) δ 193.78, 134.27, 133.90, 129.16, 128.92, 52.92, 20.13.

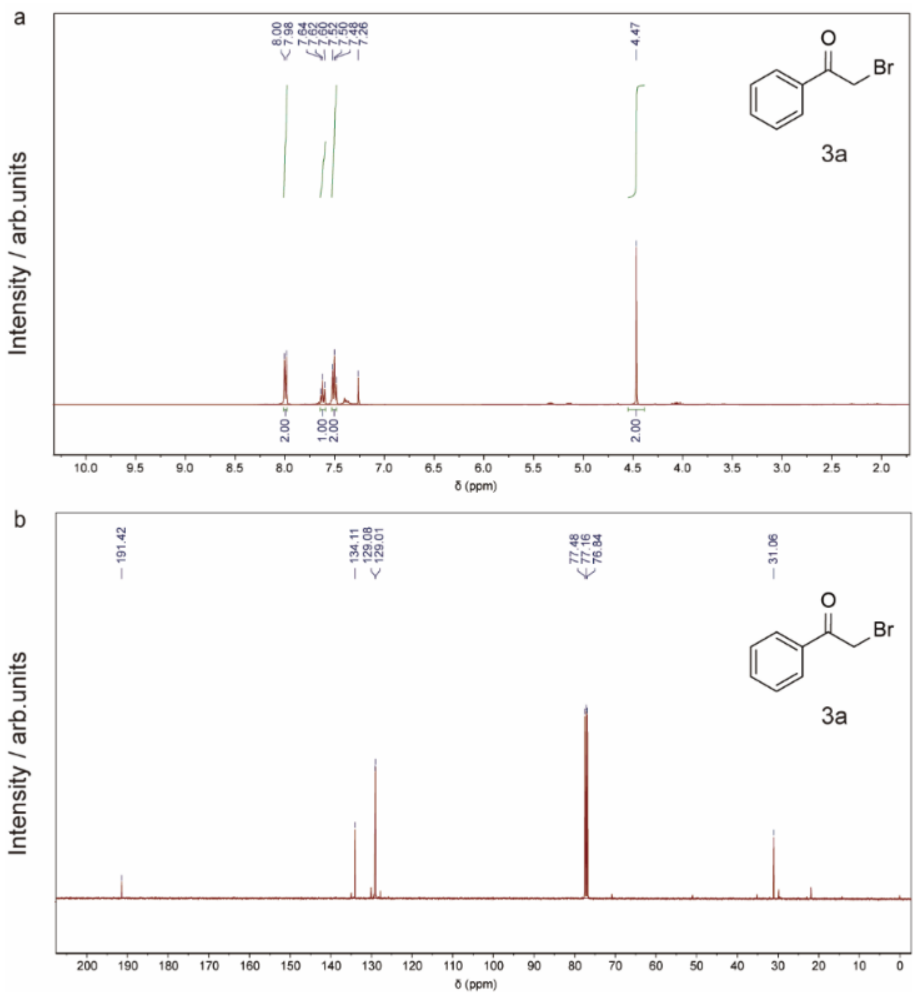

Figure S25. NMR spectra of 3a. (a) ^{1}H NMR (400 MHz, $CDCl_3$) δ 7.99 (d, J = 7.6 Hz, 2H), 7.62 (t, J = 7.4 Hz, 1H), 7.50 (t, J = 7.7 Hz, 2H), 4.47 (s, 2H). (b) ^{13}C NMR (100 MHz, $CDCl_3$) δ 191.42, 134.11, 129.08, 129.01, 31.06. The spectral data of this chemical matches previous work[7].

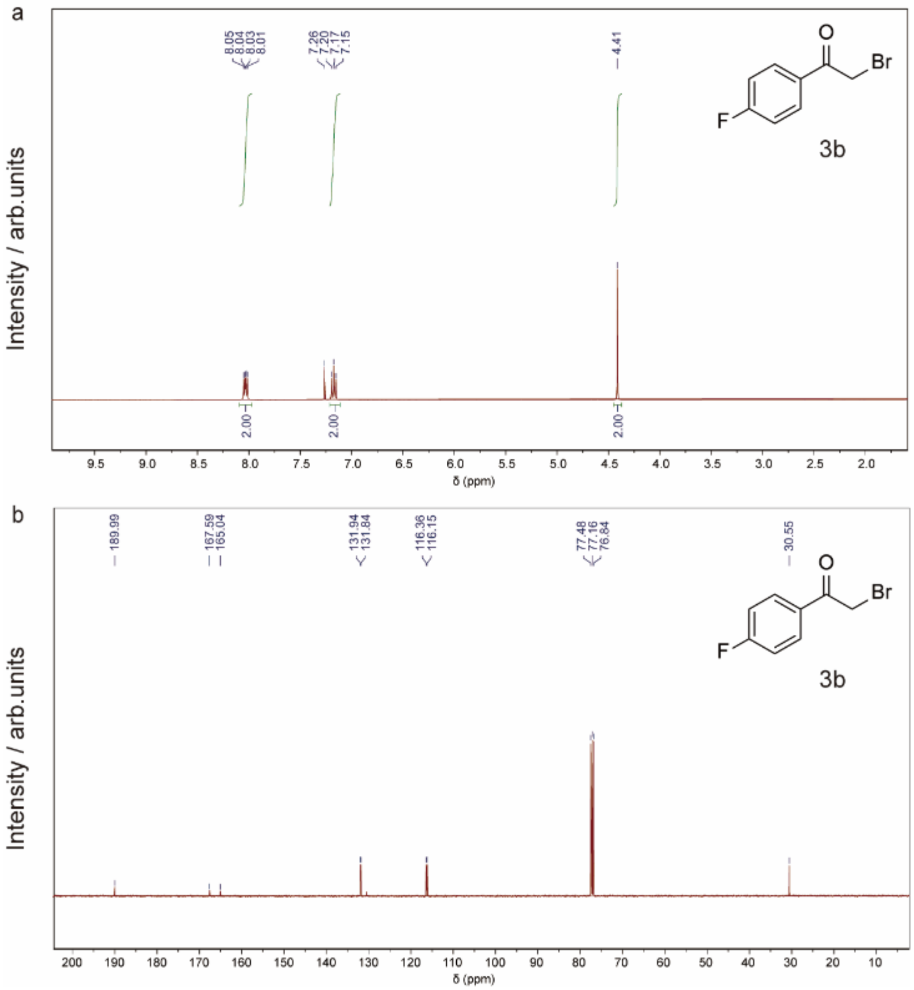

Figure S26. NMR spectra of 3b. (a) ^{1}H NMR (400 MHz, $CDCl_3$) δ 8.03 (dd, J = 8.9, 5.3 Hz, 2H), 7.17 (t, J = 8.6 Hz, 2H), 4.41 (s, 2H). (b) ^{13}C NMR (100 MHz, $CDCl_3$) δ 189.99, 167.59, 165.04, 131.94, 131.84, 116.36, 116.15, 30.55. The spectral data of this chemical matches previous work[7].

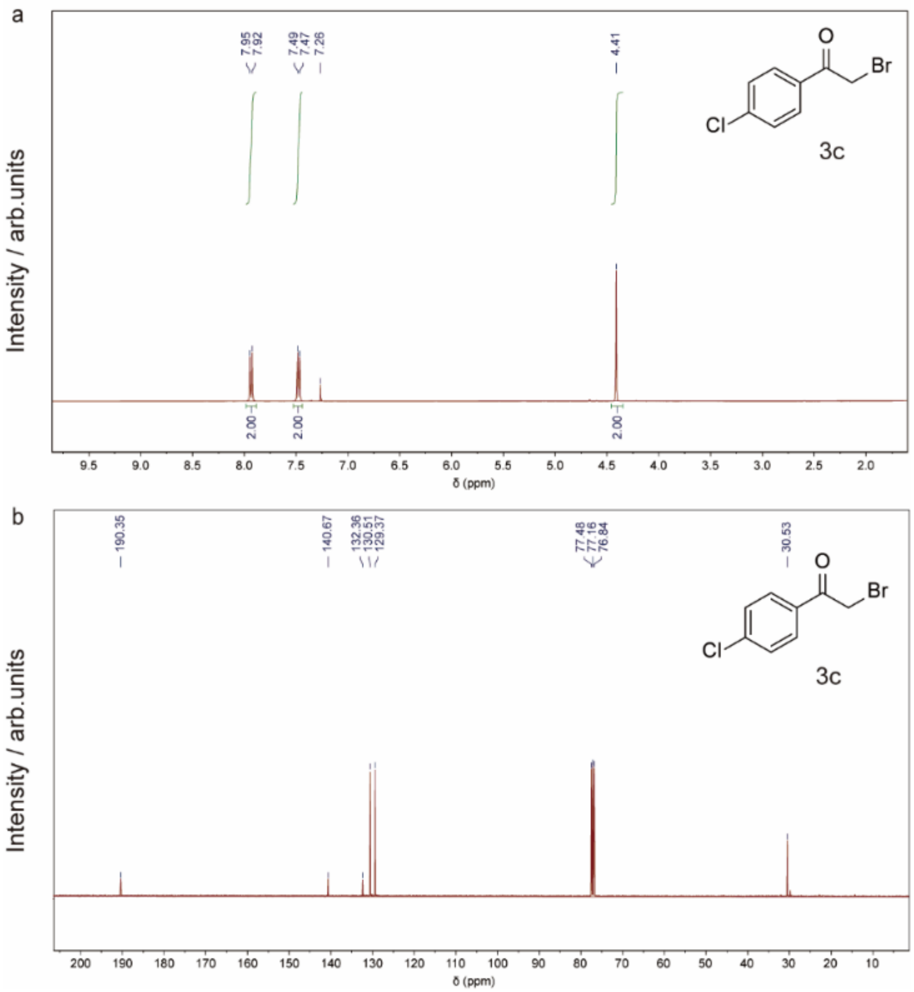

Figure S27. NMR spectra of 3c. (a) ^{1}H NMR (400 MHz, $CDCl_3$) δ 7.94 (d, J = 8.6 Hz, 2H), 7.48 (d, J = 8.6 Hz, 2H), 4.41 (s, 2H). (b) ^{13}C NMR (100 MHz, $CDCl_3$) δ 190.35, 140.67, 132.36, 130.51, 129.37, 30.53. The spectral data of this chemical matches previous work[7].

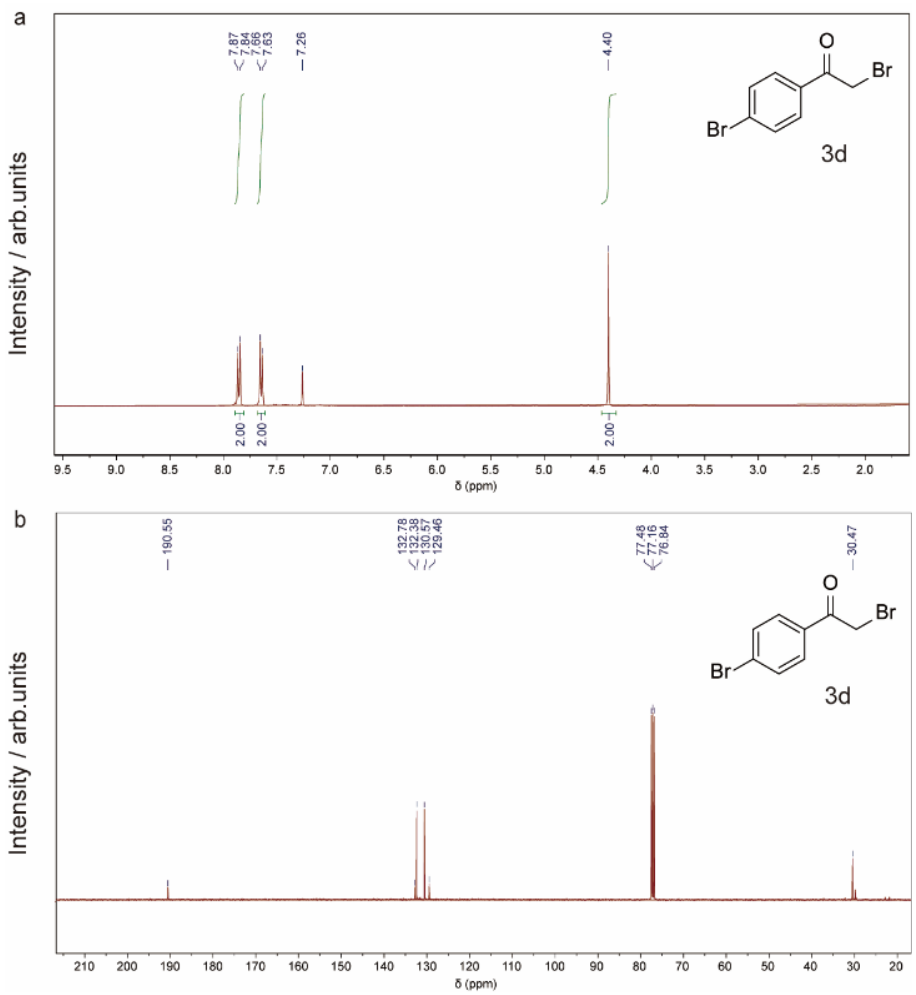

Figure S28. NMR spectra of 3d. (a) ^{1}H NMR (400 MHz, $CDCl_3$) δ 7.85 (d, J = 8.5 Hz, 2H), 7.64 (d, J = 8.5 Hz, 2H), 4.40 (s, 1H). (b) ^{13}C NMR (100 MHz, $CDCl_3$) δ 190.55, 132.78, 132.38, 130.57, 129.46, 30.47. The spectral data of this chemical matches previous work[7].

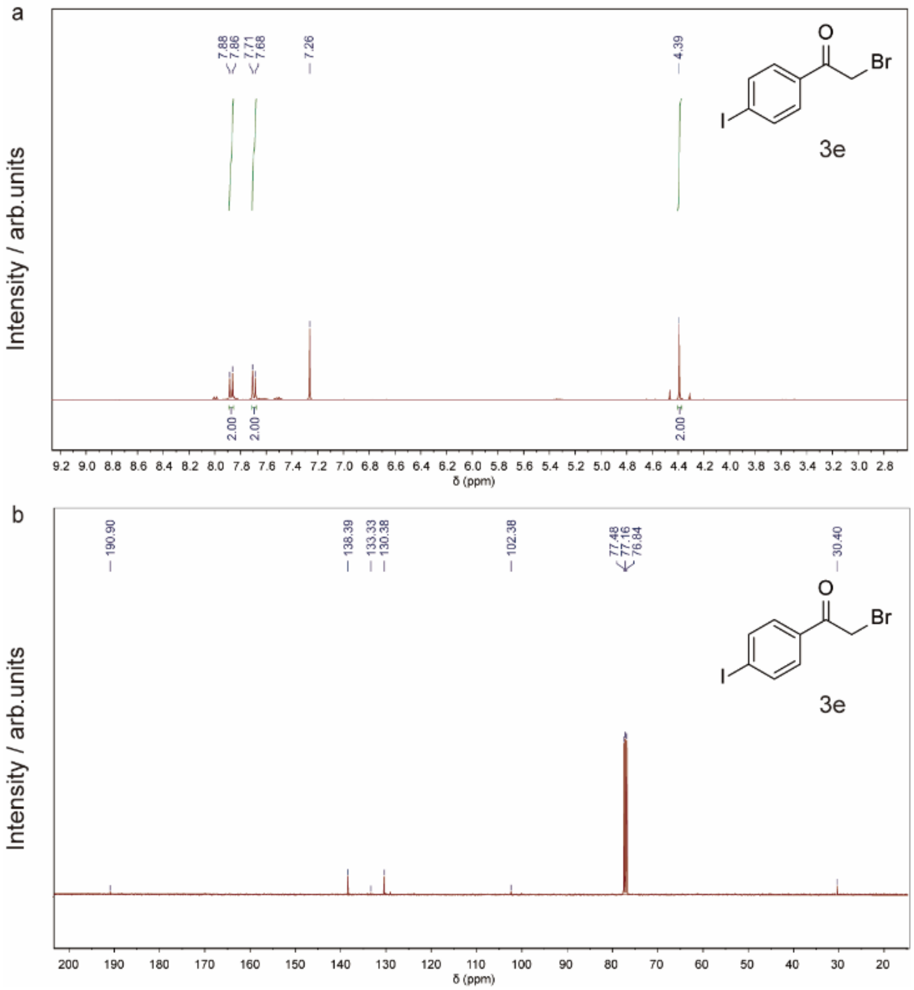

Figure S29. NMR spectra of 3e. (a) ^{1}H NMR (400 MHz, $CDCl_3$) δ 7.87 (d, J = 8.6 Hz, 2H), 7.70 (d, J = 8.6 Hz, 2H), 4.39 (s, 2H). (b) ^{13}C NMR (100 MHz, $CDCl_3$) δ 190.90, 138.39, 133.33, 130.38, 102.38, 30.40.

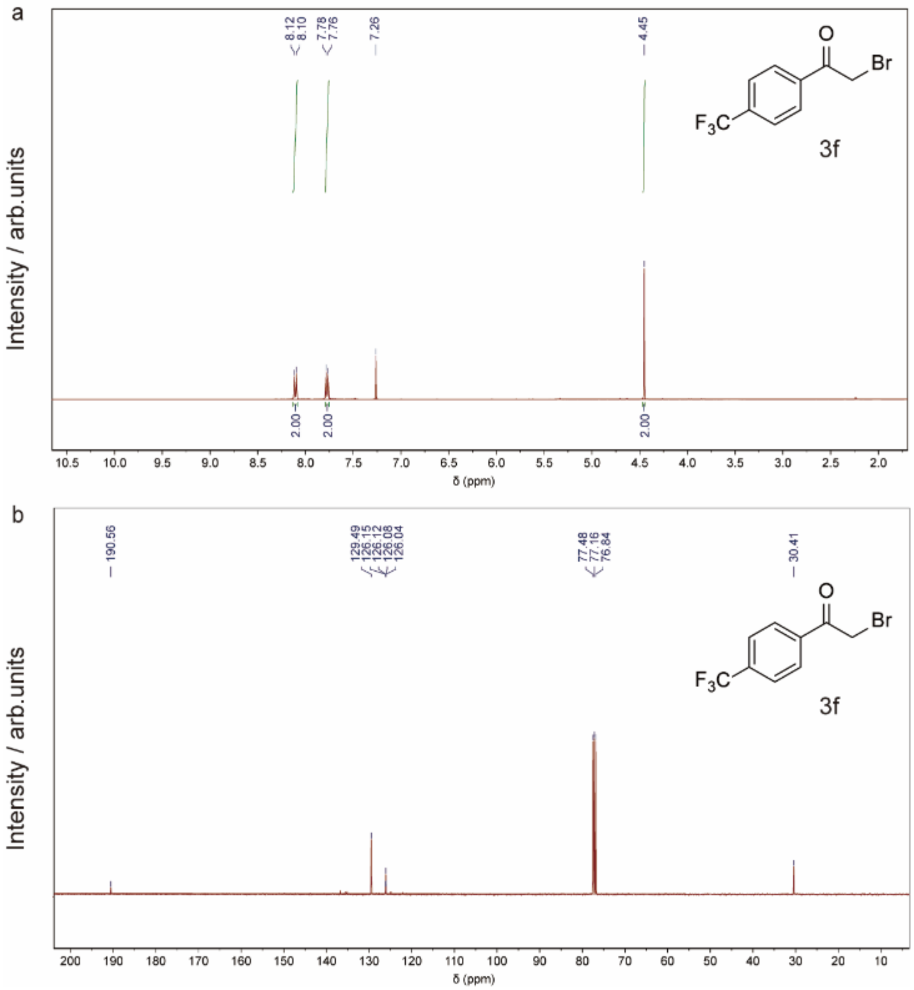

Figure S30. NMR spectra of 3f. (a) ^{1}H NMR (400 MHz, $CDCl_3$) δ 8.11 (d, J = 8.1 Hz, 2H), 7.77 (d, J = 8.2 Hz, 2H), 4.45 (s, 2H). (b) ^{13}C NMR (100 MHz, $CDCl_3$) δ 190.56, 129.49, 126.15, 126.12, 126.08, 126.04, 30.41.

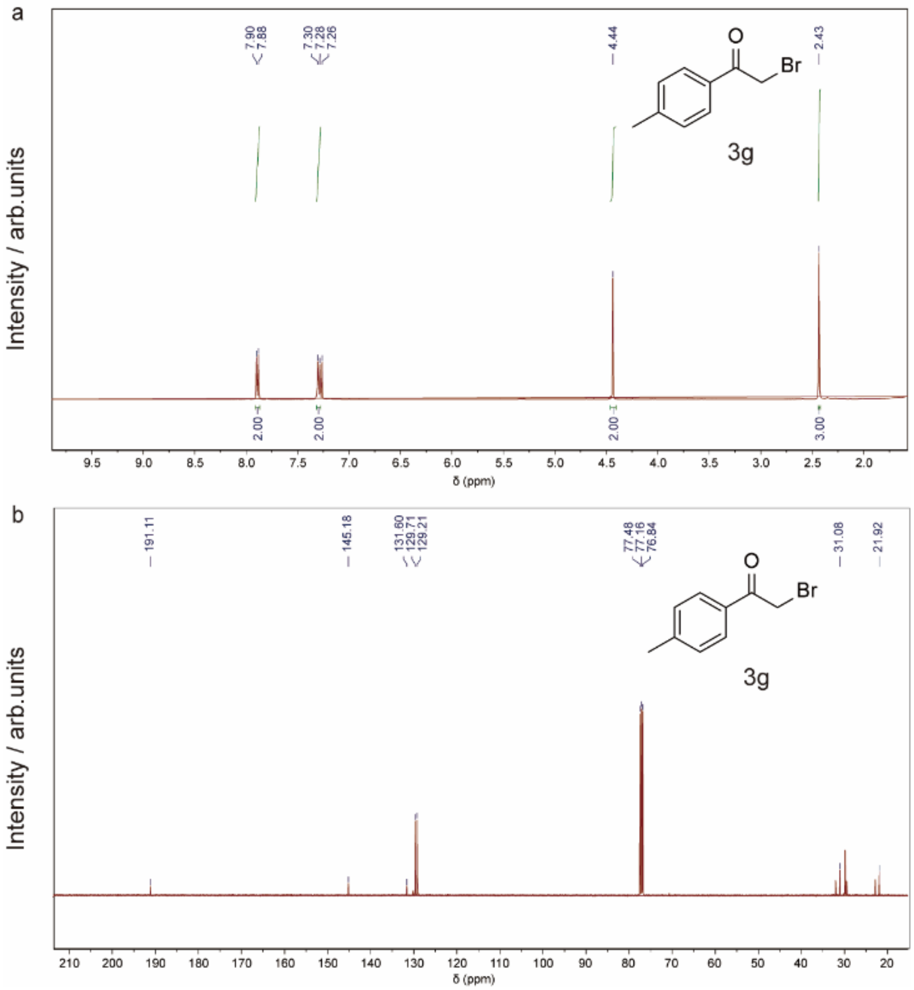

Figure S31. NMR spectra of 3g. (a) ^{1}H NMR (400 MHz, $CDCl_3$) δ 7.89 (d, J = 8.2 Hz, 2H), 7.29 (d, J = 8.1 Hz, 2H), 4.44 (s, 2H), 2.43 (s, 3H). (b) ^{13}C NMR (100 MHz, $CDCl_3$) δ 191.11, 145.18, 131.60, 129.71, 129.21, 31.08, 21.92. The spectral data of this chemical matches previous work[7].

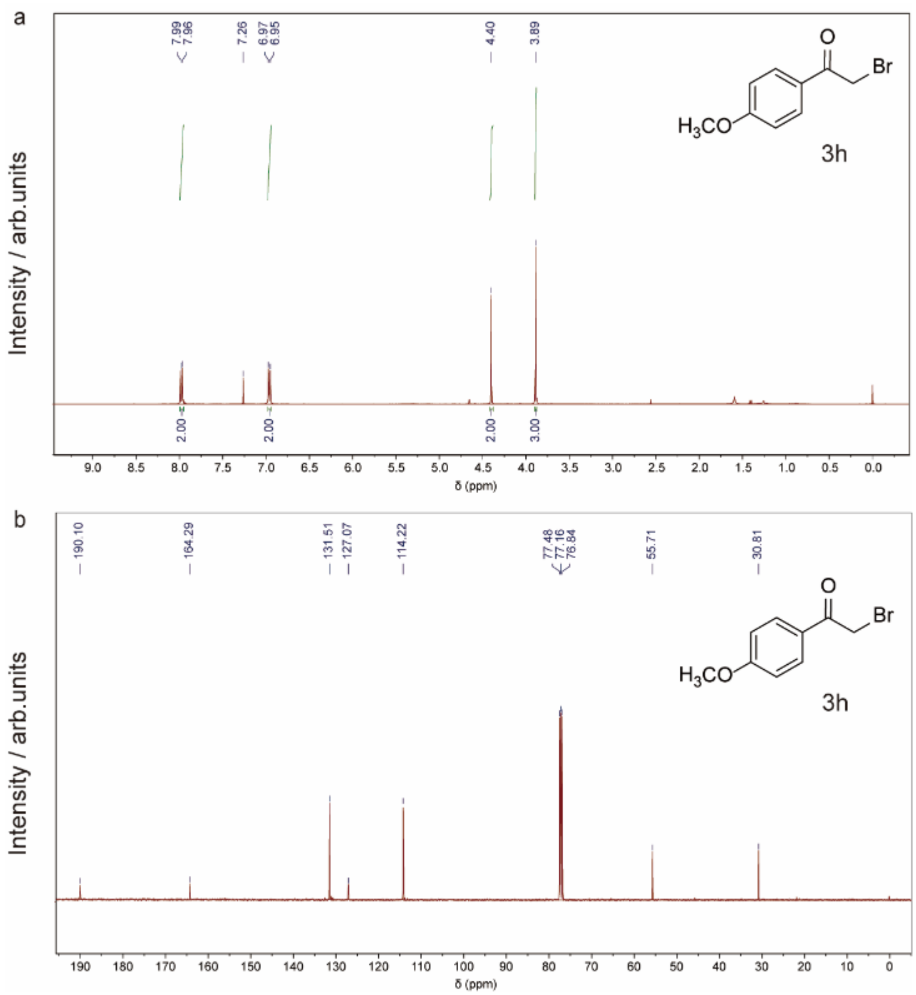

Figure S32. NMR spectra of 3h. (a) ^{1}H NMR (400 MHz, $CDCl_3$) δ 7.97 (d, J = 9.0 Hz, 2H), 6.96 (d, J = 9.0 Hz, 2H), 4.40 (s, 2H), 3.89 (s, 3H). (b) ^{13}C NMR (100 MHz, $CDCl_3$) δ 190.10, 164.29, 131.51, 127.07, 114.22, 55.71, 30.81. The spectral data of this chemical matches previous work[7].

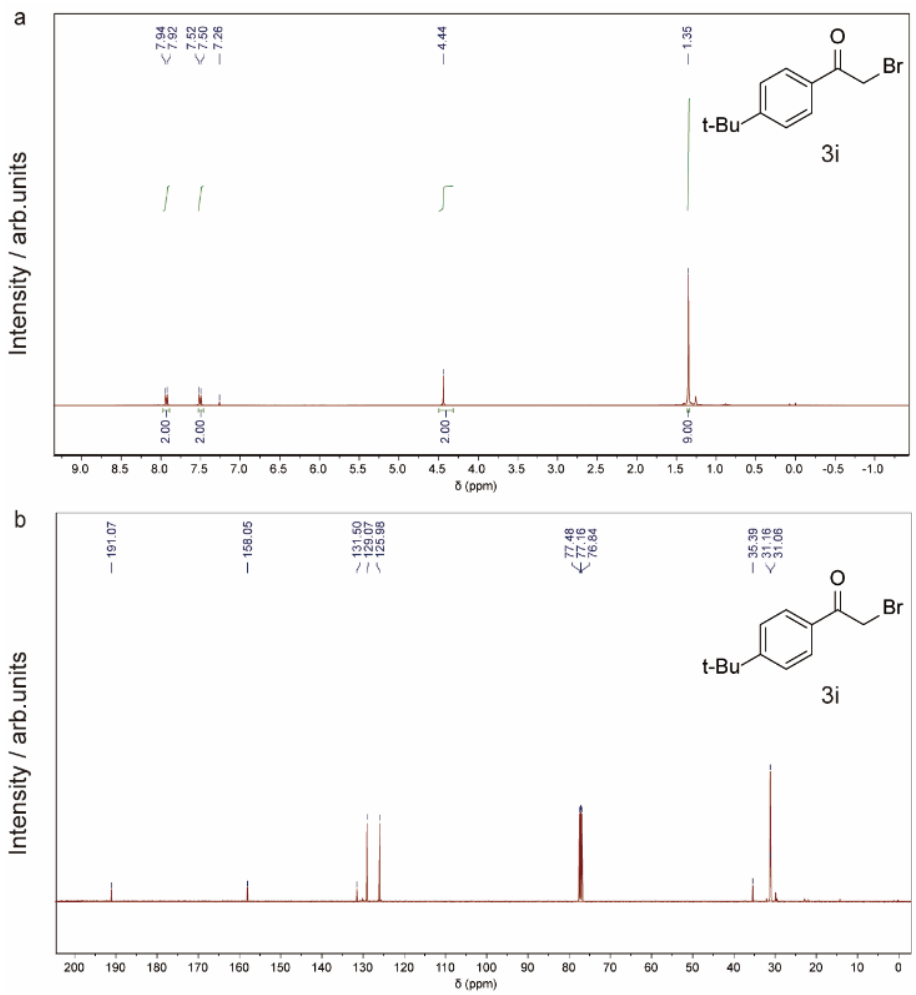

Figure S33. NMR spectra of 3i. (a) ^{1}H NMR (400 MHz, $CDCl_3$) δ 7.93 (d, J = 8.5 Hz, 2H), 7.51 (d, J = 8.5 Hz, 2H), 4.44 (s, 2H), 1.35 (s, 9H). (b) ^{13}C NMR (100 MHz, $CDCl_3$) δ 191.07, 158.05, 131.50, 129.07, 125.98, 3-5.39, 31.16, 31.06. The spectral data of this chemical matches previous work[9].

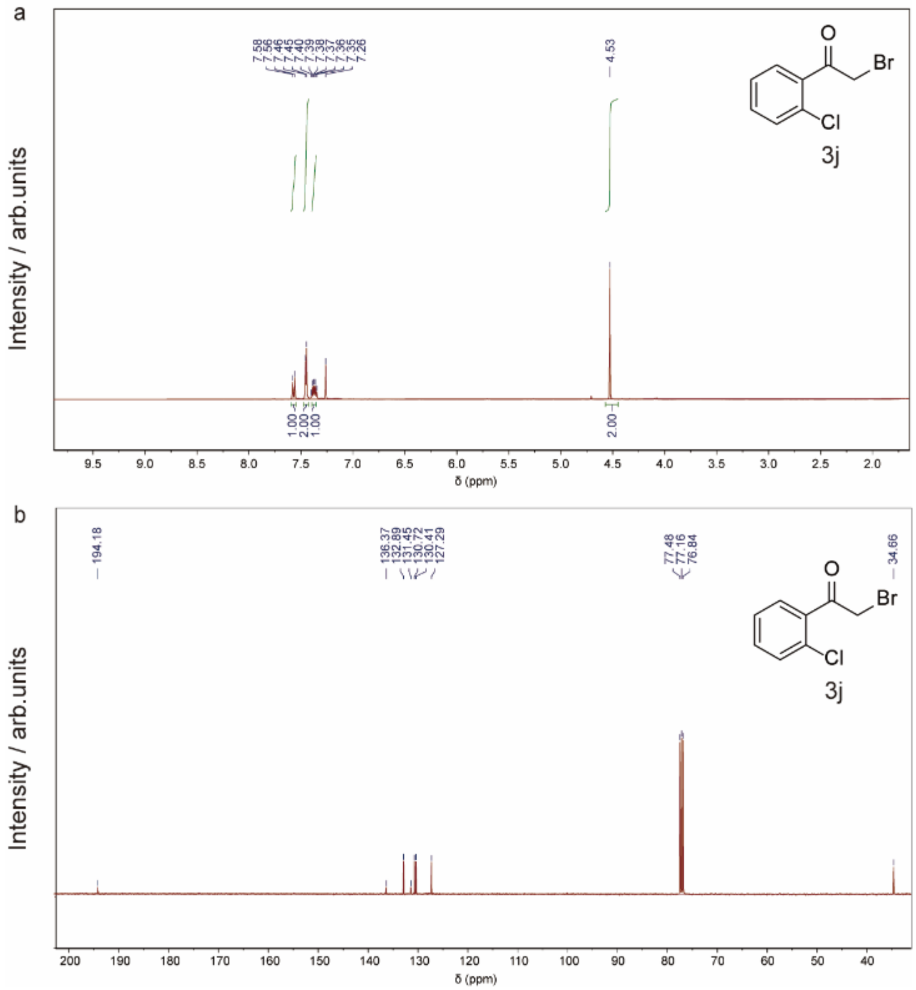

Figure S34. NMR spectra of 3j. (a) ^{1}H NMR (400 MHz, $CDCl_3$) δ 7.57 (d, J = 7.6 Hz, 1H), 7.45 (d, J = 3.8 Hz, 2H), 7.40 – 7.34 (m, 1H), 4.53 (s, 2H). (b) ^{13}C NMR (100 MHz, $CDCl_3$) δ 194.18, 136.37, 132.89, 131.45, 130.72, 130.41, 127.29, 34.66. The spectral data of this chemical matches previous work[7].

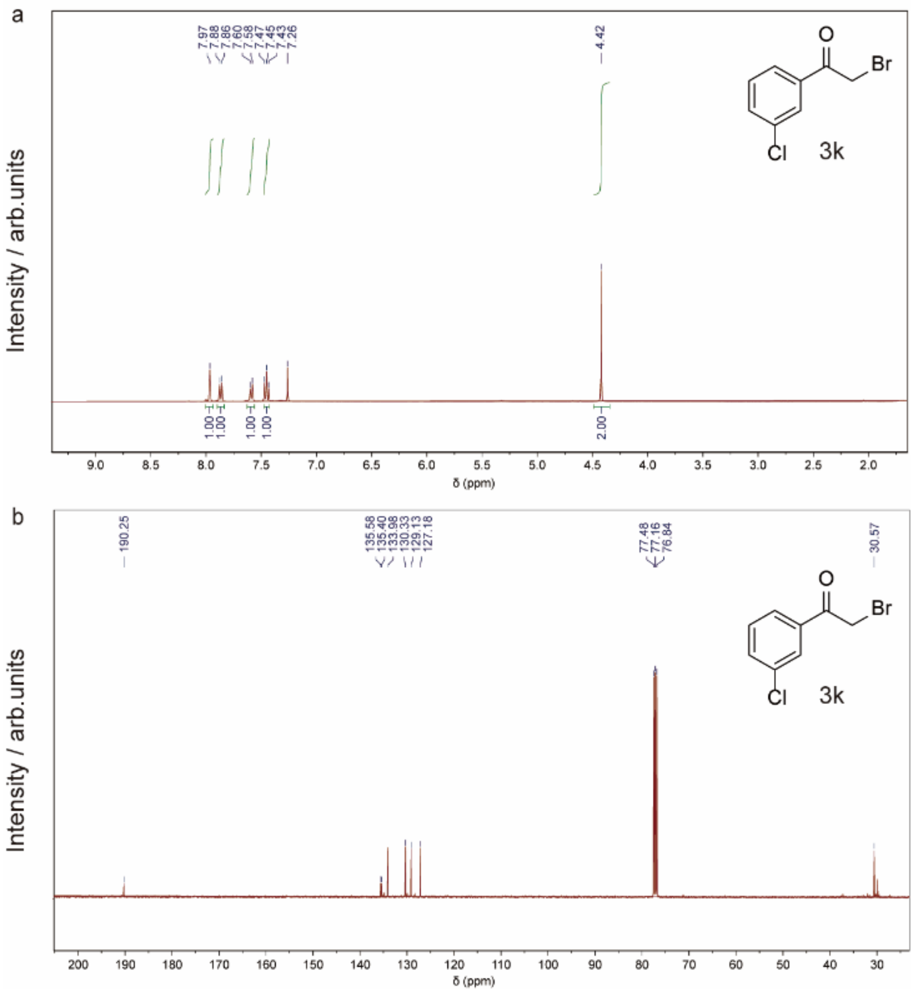

Figure S35. NMR spectra of 3k. (a) ^{1}H NMR (400 MHz, $CDCl_3$) δ 7.97 (s, 1H), 7.87 (d, J = 7.8 Hz, 1H), 7.59 (d, J = 8.0 Hz, 1H), 7.45 (t, J = 7.9 Hz, 1H), 4.42 (s, 2H). (b) ^{13}C NMR (100 MHz, $CDCl_3$) δ 190.25, 135.58, 135.40, 133.98, 130.33, 129.13, 127.18, 30.57. The spectral data of this chemical matches previous work[7].

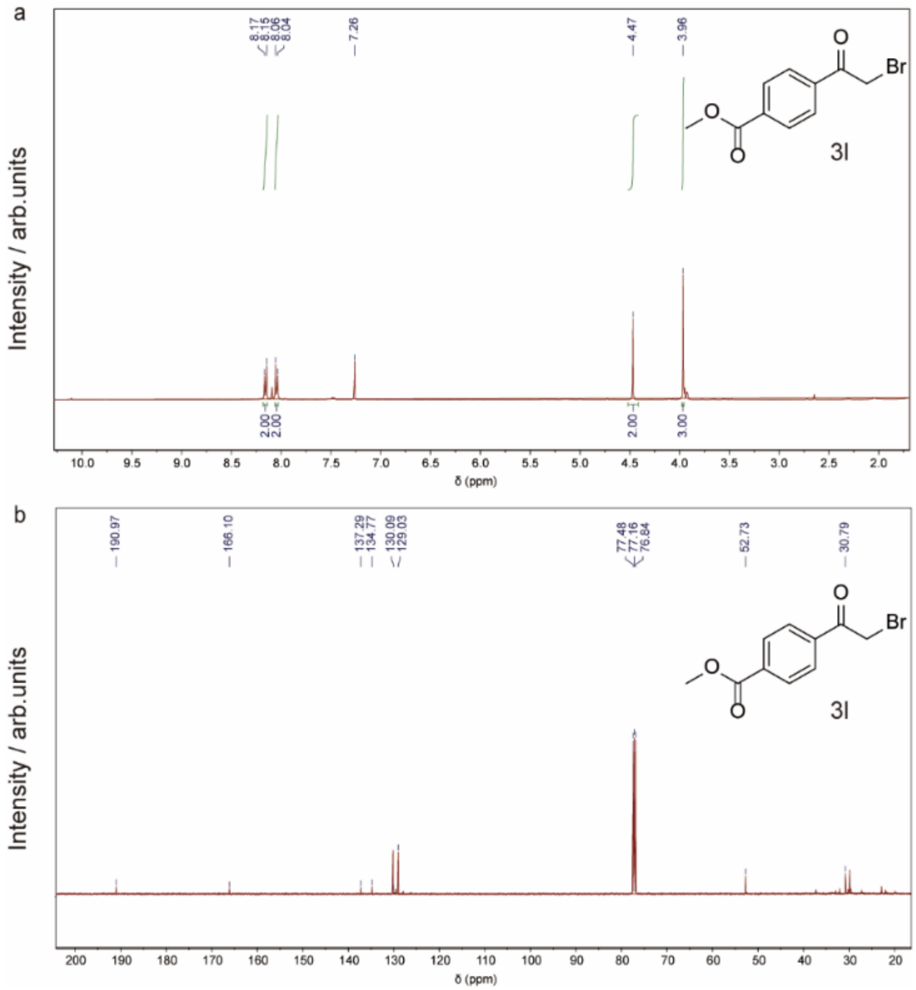

Figure S36. NMR spectra of 3l. (a) ^{1}H NMR (400 MHz, $CDCl_3$) δ 8.16 (d, J = 8.4 Hz, 2H), 8.05 (d, J = 8.4 Hz, 2H), 4.47 (s, 2H), 3.96 (s, 3H). (b) ^{13}C NMR (100 MHz, $CDCl_3$) δ 190.97, 166.10, 137.29, 134.77, 130.09, 129.03, 52.73, 30.79.

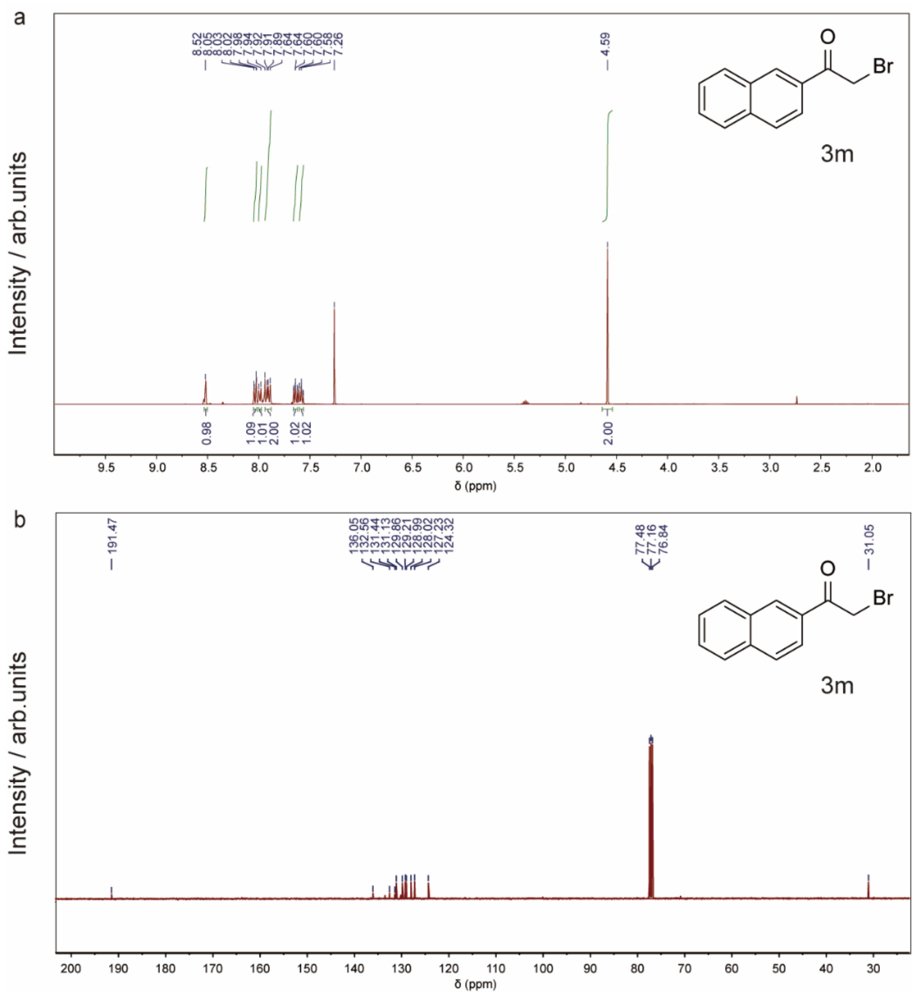

Figure S37. NMR spectra of 3m. (a) ^{1}H NMR (400 MHz, $CDCl_3$) δ 8.52 (s, 1H), 8.03 (dd, J = 8.6, 1.8 Hz, 1H), 7.99 (d, J = 8.1 Hz, 1H), 7.91 (dd, J = 12.6, 8.3 Hz, 2H), 7.67 – 7.61 (m, 1H), 7.61 – 7.56 (m, 1H), 4.59 (s, 2H). (b) ^{13}C NMR (100 MHz, $CDCl_3$) δ 191.47, 136.05, 132.56, 131.44, 131.13, 129.86, 129.21, 128.99, 128.02, 127.23, 124.32, 31.05. The spectral data of this chemical matches the previously work[8].

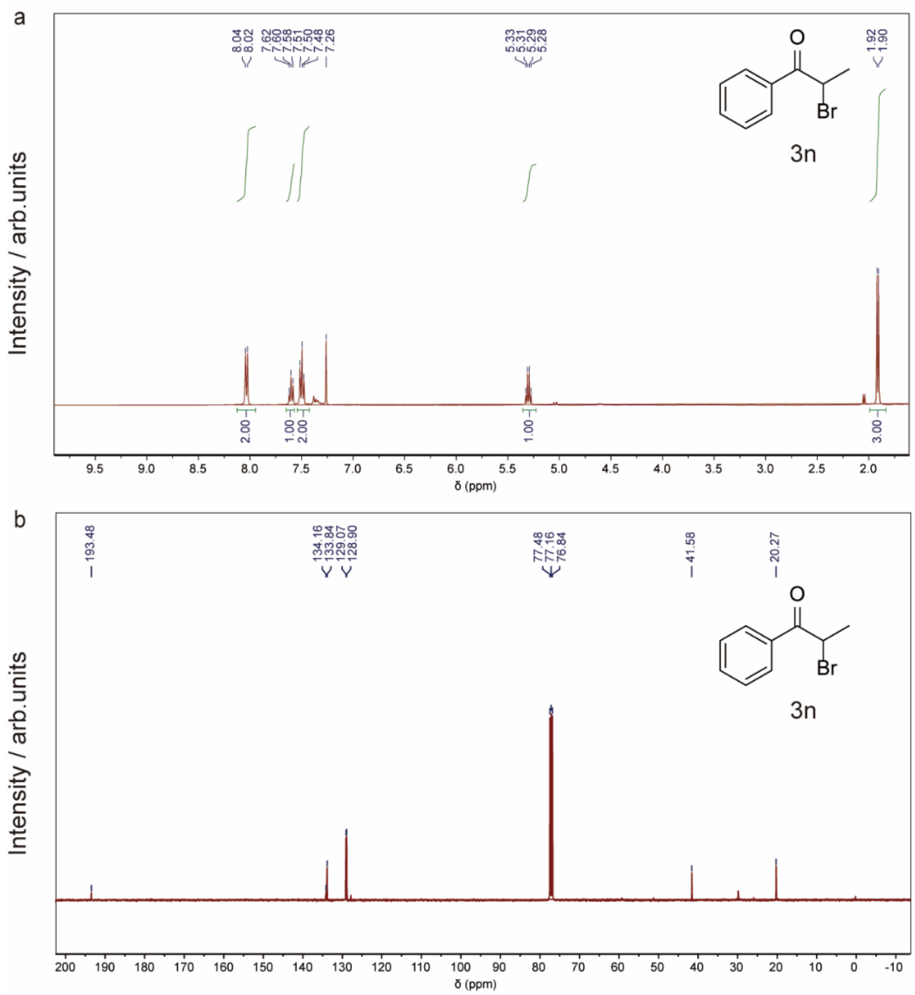

Figure S38. NMR spectra of 3n. (a) ^{1}H NMR (400 MHz, $CDCl_3$) δ 8.03 (d, J = 7.4 Hz, 2H), 7.60 (t, J = 7.4 Hz, 1H), 7.50 (t, J = 7.7 Hz, 2H), 5.30 (q, J = 6.6 Hz, 1H), 1.91 (d, J = 6.6 Hz, 3H). (b) ^{13}C NMR (100 MHz, $CDCl_3$) δ 193.48, 134.16, 133.84, 129.07, 128.90, 41.58, 20.27. The spectral data of this chemical matches the previously work[9].

Supporting Tables

Table S1. ICP-AES analysis of metal loadings on Cu-C_3N_4.

Sample	Amount of Cu	Percentage
Cu-C_3N_4	1480 μg/g	0.15%

Table S2. Control experiments of photocatalytic conversion of styrene.

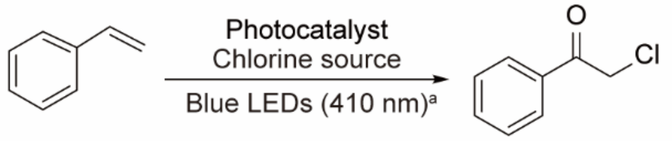

1 a → 2 a

Entry	Catalysts	Chlorine source	Solvent	Con.(%)[b]	Sel.(%)[b]
1	Ni-C_3N_4	$CuCl_2$	EtOAc-PrOH	21	0
2	N.A.	$NiCl_2 \cdot 6H_2O$	EtOAc-PrOH	23	0
3	Cu-C_3N_4	N.A.	EtOAc-PrOH	5	0
4[c]	Cu-C_3N_4	$NiCl_2 \cdot 6H_2O$	EtOAc-PrOH	0	0
5[d]	Cu-C_3N_4	$NiCl_2 \cdot 6H_2O$	EtOAc-PrOH	0	0

[a]Reaction conditions: styrene (8 mM), catalyst (50 mg), chlorine source (0.2 mmol), solvent (10 mL, 1 mL isopropanol in 9 mL ethyl acetate), RT, air, 12 h. [b]Conversion (Con.) and selectivity (Sel.) are determined by GC and GC-MS. [c]Under dark. [d]Under N_2 atmosphere.

References

(1) Jiang, L.; Yuan, X.; Pan, Y.; Liang, J.; Zeng, G.; Wu, Z.; Wang, H. Doping of graphitic carbon nitride for photocatalysis: A reveiw. *Appl. Catal., B* **2017,** *217*, 388-406.

(2) Liu, J.; Zhang, T.; Wang, Z.; Dawson, G.; Chen, W. Simple pyrolysis of urea into graphitic carbon nitride with recyclable adsorption and photocatalytic activity. *J. Mater. Chem.* **2011,** *21* (38), 14398-14401.

(3) Al-Azri, Z. H. N.; Chen, W.-T.; Chan, A.; Jovic, V.; Ina, T.; Idriss, H.; Waterhouse, G. I. N. The roles of metal co-catalysts and reaction media in photocatalytic hydrogen production: Performance evaluation of M/TiO_2 photocatalysts (M=Pd, Pt, Au) in different alcohol–water mixtures. *J. Catal.* **2015,** *329*, 355-367.

(4) Zeng, X.; Liu, Y.; Kang, Y.; Li, Q.; Xia, Y.; Zhu, Y.; Hou, H.; Uddin, M. H.; Gengenbach, T. R.; Xia, D.; Sun, C.; McCarthy, D. T.; Deletic, A.; Yu, J.; Zhang, X. Simultaneously Tuning Charge Separation and Oxygen Reduction Pathway on Graphitic Carbon Nitride by Polyethylenimine for Boosted Photocatalytic Hydrogen Peroxide Production. *ACS Catal.* **2020,** *10* (6), 3697-3706.

(5) Zhang, D.; Ren, P.; Liu, W.; Li, Y.; Salli, S.; Han, F.; Qiao, W.; Liu, Y.; Fan, Y.; Cui, Y.; Shen, Y.; Richards, E.; Wen, X.; Rummeli, M. H.; Li, Y.; Besenbacher, F.; Niemantsverdriet, H.; Lim, T.; Su, R. Photocatalytic Abstraction of Hydrogen Atoms from Water Using Hydroxylated Graphitic Carbon Nitride for Hydrogenative Coupling Reactions. *Angew. Chem. Int. Ed.* **2022,** *61* (24), e202204256.

(6) Baga, A. N.; Johnson, G. R. A.; Nazhat, N. B.; Saadalla-Nazhat, R. A. A simple spectrophotometric determination of hydrogen peroxide at low concentrations in aqueous solution. *Anal. Chim. Acta* **1988,** *204*, 349-353.

(7) Luo, Z.; Meng, Y.; Gong, X.; Wu, J.; Zhang, Y.; Ye, L.-W.; Zhu, C. Facile Synthesis of α-Haloketones by Aerobic Oxidation of Olefins Using KX as Nonhazardous Halogen Source. *Chin. J. Chem.* **2020,** *38* (2), 173-177.

(8) Wang, Z.; Wang, L.; Wang, Z.; Li, P.; Zhang, Y. A practical synthesis of α-bromo/iodo/chloroketones from olefins under visible-light irradiation conditions. *Chin. Chem. Lett.* **2021,** *32* (1), 429-432.

(9) Jiang, Q.; Sheng, W.; Guo, C. Synthesis of phenacyl bromides via $K_2S_2O_8$-mediated tandem hydroxybromination and oxidation of styrenes in water. *Green Chem.* **2013,** *15* (8), 2175-2179.
